# Moving towards a complete molecular framework of the Nematoda: a focus on the Enoplida and early-branching clades

**DOI:** 10.1186/1471-2148-10-353

**Published:** 2010-11-12

**Authors:** Holly M Bik, P John D Lambshead, W Kelley Thomas, David H Lunt

**Affiliations:** 1 Nematode Research Group, Department of Zoology, The Natural History Museum, Cromwell Road, London SW7 5BD, UK; 2 School of Ocean and Earth Science, National Oceanography Centre, European Way, Southampton SO14 3ZH, UK; 3 Hubbard Center for Genome Studies, University of New Hampshire, 35 Colovos Road, Durham, NH 03824, USA; 4 Department of Biological Sciences, University of Hull, Cottingham Road, Hull HU6 7RX, UK

## Abstract

**Background:**

The subclass Enoplia (Phylum Nematoda) is purported to be the earliest branching clade amongst all nematode taxa, yet the deep phylogeny of this important lineage remains elusive. Free-living marine species within the order Enoplida play prominent roles in marine ecosystems, but previous molecular phylogenies have provided only the briefest evolutionary insights; this study aimed to firmly resolve internal relationships within the hyper-diverse but poorly understood Enoplida. In addition, we revisited the molecular framework of the Nematoda using a rigorous phylogenetic approach in order to investigate patterns of early splits amongst the oldest lineages (Dorylaimia and Enoplia).

**Results:**

Morphological identifications, nuclear gene sequences (18S and 28S rRNA), and mitochondrial gene sequences (*cox1*) were obtained from marine Enoplid specimens representing 37 genera. The 18S gene was used to resolve deep splits within the Enoplia and evaluate the branching order of major clades in the nematode tree; multiple phylogenetic methods and rigorous empirical tests were carried out to assess tree topologies under different parameters and combinations of taxa. Significantly increased taxon sampling within the Enoplida resulted in a well-supported, robust phylogenetic topology of this group, although the placement of certain clades was not fully resolved. Our analysis could not unequivocally confirm the earliest splits in the nematode tree, and outgroup choice significantly affected the observed branching order of the Dorylaimia and Enoplia. Both 28S and *cox1 *were too variable to infer deep phylogeny, but provided additional insight at lower taxonomic levels.

**Conclusions:**

Analysis of internal relationships reveals that the Enoplia is split into two main clades, with groups consisting of terrestrial (Triplonchida) and primarily marine fauna (Enoplida). Five independent lineages were recovered within the Enoplida, containing a mixture of marine and terrestrial species; clade structure suggests that habitat transitions have occurred at least four times within this group. Unfortunately, we were unable to obtain a consistent or well-supported topology amongst early-branching nematode lineages. It appears unlikely that single-gene phylogenies using the conserved 18S gene will be useful for confirming the branching order at the base of the nematode tree-future efforts will require multi-gene analyses or phylogenomic methods.

## Background

Members of the phylum Nematoda can be found in nearly every habitat on earth, with high abundances and diverse arrays of species existing in both marine and terrestrial habitats. Nematodes are ubiquitous and integral to ecosystem functioning-they facilitate processes such as nutrient cycling, sediment stability, and even pollutant distribution in marine systems [1], yet we lack a comprehensive understanding of global diversity within this phylum. Out of an estimated 1 million to 100 million nematode species [2], fewer than 27,000 have been formally described, representing the largest taxonomic deficit for any group of animals [3,4]. This minimal sampling of nematode diversity has implications for our understanding of systematic relationships, as adequate taxon sampling has been identified as a major factor for building accurate phylogenies [5].

Marine free-living nematodes are particularly understudied compared to their terrestrial and parasitic counterparts, with only ~4,000 species known to science. The order Enoplida (subclass Enoplia) contains a diverse group of primarily marine taxa; these nematodes represent the largest marine species in terms of physical size, and can reach up to several millimetres in length [6]. Many Enoplids are thought to be active predators (due to the complex array of teeth and mandibular structures exhibited in several families), and play important ecological roles within meiofaunal communities. However, evolutionary relationships within the Enoplida are poorly understood-published phylogenies have been overly reliant on terrestrial and parasitic species, (with the exception of Meldal *et al*. [7]), and have inadequately sampled the known diversity of the Enoplida [7-10].

Before the advent of molecular techniques, a number of taxonomic classifications attempted to catalogue the substantial morphological diversity observed within the subclass Enoplia. Filipjev [11] was the first author to produce a comprehensive morphological classification of free-living nematodes, and subsequent revisions that included a focus on the Enoplia were completed by Pearse [12], Chitwood and Chitwood [13], Clark [14], De Coninck [15], Andrassy [16], Maggenti [17], Lorenzen [18], and Siddiqi [19]. Morphological schemes primarily differed in their placement of the Tripyloididae, Alaimidae, Ironidae (all currently grouped in the Enoplida), and the Mononchoidea (now grouped under the subclass Dorylaimia). Lorenzen's [18] framework is the currently accepted classification system for free-living marine Enoplids, and has been used as the basis for Platt & Warwick's [20] ubiquitous illustrated keys for identifying genera.

De Ley and Blaxter [8,21] proposed the first comprehensive classification of the Enoplia based on SSU sequence data, following on from the original molecular phylogeny by Blaxter *et al*. [22]. This framework outlined two ranks within the Enoplia, orders Enoplida and Triplonchida; seven suborders were denoted within the Enoplida (Enoplina, Oncholaimina, Ironina, Tripyloidina, Trefusiina, Campydorina and Alaimina), and three within the Triplonchida (Diphtherophorina, Tobrilina, and Tripylina). Despite this proposed classification, De Ley and Blaxter's framework was based on relatively few gene sequences compared to the most recent molecular phylogenies [10]. These subsequent frameworks also offered limited insight regarding the Enoplida, with only a few authors sequencing additional species to supplement publically available data [7,10]. In a study focused on marine species, Meldal *et al*. [7] noted that the monophyly of some Enoplid families was highly supported (e.g. the Oncholaimoidea and Tripyloididae), whilst other families were suspected to be polyphyletic (e.g. the Ironidae). Holterman *et al*. [9] and Van Megen *et al*. [10] recovered the Bastianiidae and the Rhabdolaimidae within the Enoplida, despite De Ley and Blaxter's original placement within the order Plectida (subclass Chromadoria). Van Megen *et al.'s *[10] recent phylogeny of nematodes utilised more Enoplid sequences than any prior investigation (39 sequences representing 22 genera). Despite this expansion, taxon sampling remained sparse for some groups and the placement of many major clades in the Enoplida remained unresolved.

These published phylogenies have also transformed our understanding of relationships amongst major nematode clades. However, there is continued uncertainty surrounding the basal splits within the Nematoda. Earlier frameworks identified both the Enoplia and Dorylaimia as early-branching nematode taxa [7,22], although these studies reported a polytomy at the basal node. Holterman *et al*. [9] and Van Megen *et al.'s *[10] large-scale analysis both recovered the Enoplia as the earliest-branching clade amongst nematodes, although support values for this topology were low-only 0.81 (Bayesian posterior probability) and 65% (ML bootstrap), respectively. Understanding the order of early splits is crucial for accurately reconstructing nematode evolution; molecular phylogenies provide a valuable glimpse back in time, given the non-existent fossil record for nematodes. The Enoplia (a primarily marine group) and Dorylaimia (a terrestrial group) represent very different life histories-if Dorylaimids were to be confirmed as the earliest branching lineage, it may imply a terrestrial origin for the phylum [21], and challenge the widely held view that nematodes first arose in marine environments.

Enoplids are often assumed to be the oldest nematode group [9,23], despite the continued lack of resolution in molecular phylogenies. Certain developmental traits in Enoplid species appear to represent plesiomorphic states, such as the symmetric embryonic cleavage that can be observed in *Tobrilus diversipapillatus *and several other marine species; in contrast, nematodes in other clades show asymmetric patterns that are unique amongst metazoa [4,24]. In addition, Enoplid species retain a nuclear envelope in mature spermatozoa (a plesiomorphic trait in male gamete development), compared to all other nematode groups, which show a clear loss of this structure [25-27].

There is currently little morphological and developmental evidence to insinuate ancestral traits amongst the Dorylaimia, but the absence of evidence does not preclude Dorylaimids from representing the earliest-splitting nematode clade. De Ley and Blaxter [21] present an intriguing discussion that suggests a resemblance (and potentially common ancestry) between Dorylaimid mouth structures and protrusible 'introvert' structures seen Kinorhyncha, Priapulida, and juvenile Nematomorpha. Dorylaimid species exhibit vastly different lifestyles and span a broad ecological range-this diversity has prompted speculation of an early terrestrial evolution and radiation within this group [8]; however, Dorylaimids possess surprisingly little genetic divergence despite this high diversity, which may alternatively signify a quite recent evolutionary origin for these terrestrial species. A molecular framework with firm support at the basal node will be necessary for resolving the longstanding debate about early splits within the Nematoda.

This study aimed to resolve phylogenetic relationships within the Enoplida using greatly increased taxon sampling and sequence data from multiple genes. Prior to this investigation, few publically available full-length SSU sequences were available for Enoplid nematodes. Morphological data and up to three gene sequences (18S, 28S and *cox1*) were collected from a total of 254 nematodes from the order Enoplida, representing 37 genera collected from marine habitats. Large-scale phylogenies were constructed in order to assess the overall placement of the Enoplia in the nematode framework, elucidate major clades within the previously undersampled Enoplida, and investigate lower taxonomic relationships amongst Enoplid genera.

## Results

### Resolving relationships within the Enoplida

The 18S rRNA gene and the D2/D3 region of the 28S rRNA gene were both amplified from a total of 254 Enoplid nematodes, and *cox1 *was additionally isolated from a subset of 99 specimens. All gene sequences were subsequently deposited in GenBank (Accession Numbers listed in Additional file 1). Amongst different phylogenetic topologies, clade membership and lower taxonomic relationships within the Enoplida were consistent between trees. Congruent topologies were obtained using both small and large 18S datasets (comprised of Enoplid taxa only and all nematode taxa, respectively). Certain higher clade relationships in the Enoplida remained unresolved; the placement of the *Syringolaimus*/*Campydora/*Rhabdolaimidae and Ironidae/Alaimina clades were often unstable, and the order of sub-clades within the Oncholaimoidea/Oxystominidae group, often changed between trees. In this study, both 28S and *cox1 *were too variable for inferring deep phylogeny. Alignments of 28S sequences showed regions of high variability and uncertain orthology between divergent groups and consequently higher clade relationships did not always correspond well with 18S data. Other authors have used these genes to elucidate relationships at the genus level or below [28-30], and in our analyses lower taxonomic relationships inferred from 28S data largely agreed with 18S topologies. In a few cases, 28S added further insight to relationships amongst genera compared to 18S phylogenies (e.g. within the family Tripyloididae). Fewer inferences were possible from the limited *cox1 *dataset, but many genus-level relationships corresponded well with 28S and 18S tree topologies.

### A molecular framework of the Enoplia

**Figure 1:**
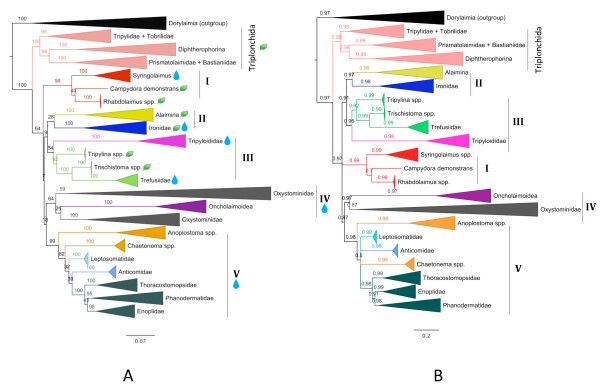
**Maximum Likelihood and Bayesian 18S phylogenies of the Enoplia. **Maximum Likelihood and Bayesian phylogenies of the Enoplia, outlining major clades within the Triplonchida and Enoplida. (A) Majority-rule ML Tree built using 18S sequence data from 381 taxa, with estimation of the P-Invar parameter and partitions according to 18S secondary structure. Pictograms denote marine (blue drop) and terrestrial/freshwater (green leaf) lineages within the Enoplida. (B) Bayesian tree built using 18S sequence data from 381 taxa, using the GTR+G+I model of nucleotide substitution and partitions according to 18S secondary structure.

The Enoplia is divided into two orders, the Triplonchida and the Enoplida (Figure 1). This separation is highly supported in ML and Bayesian analyses, and agrees with previous molecular frameworks [8-10]. The Triplonchida was consistently recovered as monophyletic, in line with previous phylogenies [7-9]; this group contained three well supported sub-clades (bootstraps >94% and posterior probabilities >0.98), and arrangement of taxa within this group agrees with the most recent nematode phylogeny by Van Megen *et al*. [10]. Unfortunately, online sequence databases did not contain many 28S sequences from Enoplid nematodes, so it was not feasible to clarify lower taxonomic relationships for terrestrial clades within the Triplonchida-only marine taxa were sequenced during this study.

ML and Bayesian analyses recovered five distinct lineages within the order Enoplida (Figure 1). Clade I contains only the Rhabdolaimidae, the genus *Syringolaimus *and *Campydora demonstrans*. Clade II contains the Alaimina and Ironidae (excluding *Syringolaimus*) as sister taxa. The Tripyloididae and the Trefusiidae (including the genera *Tripylina *and *Trischistoma*) were recovered as sister taxa in Clade II. Clade IV contained both the Oxystominidae and the superfamily Oncholaimoidea. Finally, the genera representing the superfamily Enoploidea *sensu *Lorenzen [18] plus the Leptosomatidae represented Clade V within the Enoplida.

The Oxystominidae and superfamily Oncholaimoidea (Clade IV, Figure 1) were always recovered as a monophyletic grouping in tree topologies, although the relationships between sub-clades are not fully resolved in either ML or Bayesian topologies. Support values for the Oncholaimoidea-Oxystominidae clade were not always high in ML analyses (ranging from 50-75%), but this grouping was recovered with high support in Bayesian topologies (>0.97). The Oncholaimoidea is firmly supported as monophyletic in 18S, 28S and *cox1 *tree topologies (support values of 100% and 0.97) and includes both the Oncholaimidae and the Enchelidiidae, in line with previous phylogenies [7,8,10]. The Enchelidiidae appears to be a more recently derived clade within the Oncholaimoidea; this family is consistently recovered as a monophyletic, although support values vary (ML 55-75% and Bayesian 0.95). The family Oncholaimidae is confirmed to be paraphyletic, as first suggested by Van Megen *et al*.'s [10] topology.

Within the Oncholaimidae, the genera *Oncholaimus *and *Viscosia *appear to exhibit substantial molecular diversity. According to genetic data, most shallow water *Oncholaimus *specimens were placed in a clade next to *Viscosia*; the primary shallow-water clades containing the majority of *Oncholaimus *and *Viscosia *specimens isolated in this study are denoted by a black square in Additional file 2, Figure S1. However, there also appeared to be another divergent group of *Oncholaimus *nematodes within the Oncholaimoidea. Two shallow water sequences obtained from GenBank, *Oncholaimus spp*. and *Viscosia sp*., appeared more closely related to the divergent *Oncholaimus *group containing deep-sea specimens, and not to the main clades representing specimens from intertidal sediments.

The placement of genera within the Oxystominidae is not overly stable in 18S trees, and this group is sometimes recovered as paraphyletic (Clade IV, Figure 1a). The four sub-clades (Oncholaimoidea, *Thalassoalaimus/Cricohalalaimus/Litinium*, *Oxystomina*, and *Halalaimus*) were observed to vary consistently in regard to their internal placement and splitting order. The genus *Halalaimus *is consistently recovered as a long-branch clade, and it is possible that this taxon has a destabilizing effect on the internal topology of the Oncholaimoidea/Oxystominidae clade.

This study always recovered the families Tripyloididae and Trefusiidae within a single clade (Clade III, Figure 1), despite varying support values (ML 54-82% and Bayesian 0.96); this clade also contains two terrestrial genera formerly grouped within the Tripylidae, *Tripylina *and *Trischistoma*. Molecular evidence has firmly placed the terrestrial Tripylidae family within the Triplonchida, and not within the Enoplida; *Tripylina *and *Trischistoma *are strongly supported (ML usually >90% and Bayesian 0.92) within the Enoplida, suggesting that their past classification within the Tripylidae was based on homoplasious morphological characters.

Large subunit sequences were able to add further resolution regarding relationships within the Tripyloididae. Data from 18S suggest that the genera *Bathylaimus *and *Tripyloides *do not form distinct, separate lineages (Additional file 2, Figure S1). Maximum Likelihood phylogenies built using 28S data from the same Enoplid specimens (Additional file 2, Figure S2) clearly differentiate one genus from the other and denote a sister relationship for these two taxa. However, mitochondrial trees (Additional file 2, Figure S3) resemble 18S topologies-*cox1 *does not indicate a clear distinction between genera, as indicated by 28S data.

The proposed monophyly of the superfamily Enoploidea [7,8] is not upheld by the present study. The Enoploidea *sensu *Lorenzen [18] instead appears to be paraphyletic, with all genera recovered in a well supported clade that additionally included the Leptosomatide (Clade V, Figure 1). This large grouping contains the Enoplidae, Thoracostomopsidae, Anoplostomatidae, Phanodermatidae, Anticomidae, and Leptosomatidae. The Anoplostomatidae *sensu *Lorenzen [18] (comprising the genera *Anoplostoma *and *Chaetonema*) appears to be polyphyletic; the genus *Anoplostoma *is an early branching clade within the Enoploidea, while the genus *Chaetonema *occupies a more derived position. The Leptosomatidae was not extensively sampled (only two gene sequences representing genera *Leptosomatides *and *Syonchus *were obtained during this study), but specimens from this family were always recovered within a single clade with high support (100% bootstrap support in ML and posterior probabilities of 0.99 in Bayesian trees). The Anticomidae was also recovered as monophyletic. The branching order of clades within the Enoploidea was unresolved in Bayesian trees and exhibited low support in ML topologies; denser taxon sampling within these families may help to resolve the exact branching order in future studies. The Thoracostomopsidae, Enoplidae and Phanodermatidae were always recovered within a monophyletic clade (ML 100% and Bayesian 0.99). The Phanodermatidae and Enoplidae were usually recovered as sister taxa, although support values are low under ML analysis (40-50%) and not especially high in Bayesian topologies (0.91).

Both 18S and 28S data suggest that species within the Phanodermatidae are morphologically similar but genetically diverse. In this study, most specimens identified within this group had few distinguishing features; ribosomal sequence data confirms the monophyly of the Phanodermatidae, but tree topologies appear to divide the family into three distinct clades (Additional file 2, Figure S1). The observed clustering of taxa was identical in both 18S and 28S tree topologies. Mitochondrial trees did not resolve the Phanodermatidae as monophyletic (possibly due to the inclusion of fewer mitochondrial sequences and/or the presence of divergent haplotypes), but *cox1 *trees additionally support the grouping of taxa observed in ribosomal phylogenies (Additional file 2, Figure S3).

The divergent clade containing the Rhabdolaimidae, the genus *Syringolaimus*, and *Campydora demonstrans *was recovered with high support (ML 98% and Bayesian 0.99) in 18S topologies (Clade I, Figure 1). The Rhabdolaimidae was consistently recovered as a sister taxon to *Campydora demonstrans*, despite low support values (ML < 50% and Bayesian 0.70). Previous studies insinuated a close relationship between *Syringolaimus *(formerly grouped within the Ironidae) and *Campydora *[7,9,10], and our results uphold these findings. Relationships amongst other species in the Ironidae remain unresolved, although it appears that at least several genera form a monophyletic grouping. *Dolicholaimus *and *Ironus *were consistently recovered as single clade with high support (ML 100% and Bayesian 0.98). This study was only able to obtain gene sequences from two Ironidae genera; additional sampling will be required to resolve placement of other taxa within this group.

The Alaimina was recovered in accordance with Van Megen *et al*. [10], and was often observed to form a sister relationship with the Ironidae (Clade II, Figure 1). This clade arrangement showed minimal support in ML topologies, but Bayesian trees recovered high support values for this Alaimina/Ironidae association (0.97).

### Resolving the order of early branching nematode clades

The large-scale Maximum Likelihood topologies recovered in this study agree broadly with previously published nematode phylogenies [7,9,10], recovering all major nematode clades and providing increased resolution at certain nodes. We found good agreement across optimality criteria with both Bayesian and Maximum Likelihood methods; our analyses revealed that the Dorylaimia and Enoplia split early from other nematode lineages, although tree topologies did not definitively resolve the branching order of these two groups.

**Figure 2:**
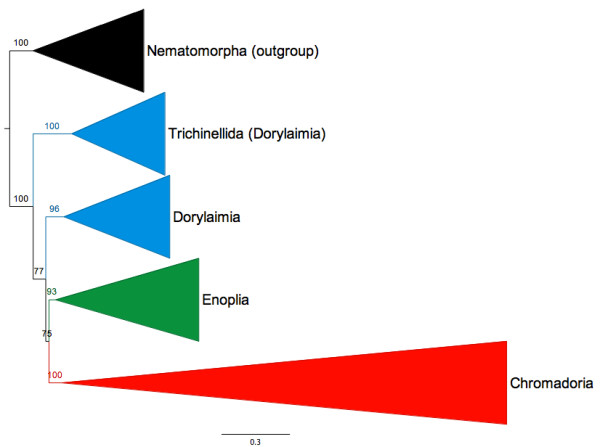
**Maximum Likelihood 18S phylogeny recovering the Dorylaimia as the earliest splitting lineage. **An example of a Maximum Likelihood 18S topology recovering the Dorylaimia as the earliest splitting nematode clade. Tree built using 1355 taxa (including Nematomorpha as outgroup taxa), estimation of the P-Invar parameter, and partitions according to 18S secondary structure.

Three scenarios (all with low support) were observed across ML tree topologies: 1) the Dorylaimia splitting first from all other nematodes (Figure 2), 2) the Enoplia splitting first from all other nematodes, or 3) the Dorylaimia and Enoplia placed as sister taxa in a single clade that appears as a sister taxon to all other nematodes. The first and second scenarios were the most common across different parameters and outgroup taxa (Table 1); the third scenario was only rarely recovered. The choice of outgroups and phylogenetic parameters had a significant impact on this branching order of nematode clades (summarized in Table 1). The Dorylaimia was always observed to split off first when nematode taxa were analysed alongside the purportedly closest metazoan relative, the Nematomorpha (Figure 2). The Enoplia was repeatedly observed to split off at the basal node using either the Priapulida or Kinorhyncha as outgroups, but when these same datasets were partitioned according to secondary rRNA structure the Dorylaimia was instead recovered as the earliest branching clade. The Tardigrade outgroup usually recovered the Dorylaimia splitting off at the basal node, although occasionally the Enoplia and Dorylaimia were recovered as sister taxa. Adding a second, third, or fourth outgroup to the dataset did not improve resolution at the base of the tree, and the Dorylaimia and Enoplia were alternately recovered as the earliest branching nematode lineage. When four different non-nematode phyla were included in gene alignments (representing a mix of close relatives and more distant taxa according to the phylogenies proposed in Dunn *et al*. [31]), the Enoplia was usually observed as the earliest-splitting lineage. However, when the same four non-nematode phyla were used in conjunction with Gblocks analysis (thus removing divergent or saturated alignment positions), the Dorylaimia was instead recovered as the earliest branching clade.

**Table 1:** Characterising the earliest splits in the maximum likelihood phylogeny of Nematoda

Nematode Taxa and Treatment	Outgroup	Additional Non-nematode Phyla	Earliest Splitting Clade	Bootstrap Support for Earliest Split
All Taxa	Priapulida+Kinorhyncha	Tadigrada, Nematomorpha	Enoplia	19%

All Taxa	Kinorhyncha	(none)	Dorylaimia*	41%

All Taxa	Nematomorpha	(none)	Dorylaimia*	75%

All Taxa	Priapulida	(none)	Dorylaimia*	45%

All Taxa	Tardigrada	(none)	Dorylaimia*	49%

All Taxa (No P-invar estimate)	Priapulida+Kinorhyncha	Tardigrada, Nematomorpha	Enoplia	19%

All Taxa (No P-invar estimate)	Kinorhyncha	(none)	Enoplia	34%

All Taxa (No P-invar estimate)	Priapulida	(none)	Enoplia	29%

All Taxa (No P-invar estimate)	Nematomorpha	(none)	Dorylaimia*	76%

All Taxa (No P-invar estimate)	Tardigrada	(none)	Doryla+Enop	20%

All Taxa (No P-invar estimate)	Tardigrada	Nematomorpha	Dorylaimia*	60%

All Taxa (No P-invar estimate)	Kinorhyncha	Nematomorpha	Enoplia	21%

All Taxa (No P-invar estimate)	Priapulida	Nematomorpha	Doryla+Enop*	39%

All Taxa (No P-invar estimate)	Kinorhyncha	Tardigrada	Enoplia	24%

All Taxa (No P-invar estimate)	Priapulida	Tardigrada	Doryla+Enop*	55%

All Taxa (No P-invar estimate)	Kinorhyncha+Priapulida	(none)	Doryla+Enop*	47%

All Taxa	Kinorhyncha+Priapulida	Tardigrada	Doryla+Enop*	61%

All Taxa	Kinorhyncha+Priapulida	Nematomorpha	Enoplia	32%

All Taxa	Priapulida	Tardigrada, Nematomoprha	Enoplia	19%

All Taxa	Kinorhyncha	Tardigrada, Nematomoprha	Enoplia	30%

All Taxa, Gblocks alignment	Priapulida+Kinorhyncha	Tardigrada, Nematomorpha	Dorylaimia	35%

All Taxa, Gblocks alignment	Nematomorpha	(none)	Dorylaimia*	71%

Trichinellida Removed	Priapulida+Kinorhyncha	Tardigrada, Nematomorpha	Doryla+Enop	48%

Trichinellida Removed	Kinorhyncha	(none)	Enoplia	57%

Trichinellida Removed	Nematomorpha	(none)	Dorylaimia	76%

Trichinellida Removed	Priapulida	(none)	Enoplia	54%

Trichinellida Removed	Tardigrada	(none)	Doryla+Enop	53%

Long Branch, Outlier taxa removed	Priapulida+Kinorhyncha	Tardigrada, Nematomorpha	Enoplia	25%

Long Branch, Outlier taxa removed	Nematomorpha	(none)	Dorylaimia*	69%

Basal split recovered using different combinations of nematode taxa, outgroup taxa, and phylogenetic parameters; all topologies utilized stem/loop gene partitions according to rRNA secondary structure. "Earliest splitting clade" identifies the group that diverges most basally from all other nematodes. "Doryla+Enop" indicates the Dorylaimia and Enoplia as a monophyletic group. Outgroup taxa were chosen according to the phylogeny of animal phyla by Dunn *et al*. (2008). Asterisk (*) denotes topologies where the Trichnellida and Dorylaimida are recovered as paraphyletic, with Trichnellida splitting first in all cases.

The Trichinellida (a group of animal parasites) was often recovered as a sister taxon to all other Dorylaimid species, but occasionally these two clades were resolved as paraphyletic (Table 1); previous molecular frameworks have supported the monophyly of the Dorylaimia [9,10]. The frequent paraphyly of Trichinellida in this study suggests that more intensive sampling of the diversity of this order and the Dorylaimida may be required to fully understand their systematic affinities. Removal of the Trichinellida did not result in an unequivocal basal split-outgroup choice continued to dictate the order of splits at the base of the tree. Bayesian phylogenies did not recover the Trichinellida and Dorylaimia within a monophyletic clade and did not resolve the branching order of early-splitting nematode lineages.

We tested support for the alternative split patterns at the base of the nematode phylogeny using the Shimodaira-Hasegawa log likelihood ratio test [32]. This test could not reject the hypothesis that a tree with Dorylaimida and Trichinellida as a monophyletic group was equally good as the paraphyly of these groups. A topology containing Enoplia as part of the basal split was found to be significantly worse (at the 5% level) to a basal branching of paraphyletic Dorylaimia. Yet if Dorylaimia was constrained to be monophyletic rather than paraphyletic then Enoplia branching first was not found to be a significantly worse topology.

## Discussion

### A revised framework for the Enoplia

This study represents the first comprehensive phylogeny of the nematode order Enoplida, a group of primarily marine free-living species; our investigation has obtained the first gene sequences from some Enoplid genera (e.g. *Chaetonema, Bathyeurystomina, Pareurystomina*) and even families (Leptosomatidae) that were formerly absent from public sequence databases. The resulting phylogeny has significantly resolved the molecular framework of the Enoplia as first outlined by De Ley and Blaxter [8,21], elucidating five major clades within the order Enoplida and providing substantial insight regarding relationships amongst lower taxonomic levels. Most notably, our analyses firmly place the Leptosomatidae within the superfamily Enoploidea (in line with many early morphological classifications [13-15,17]), while the Oxystominidae is consistently recovered in a major clade alongside the Oncholaimoidea; previous frameworks incorrectly placed both these families within the Ironina [8,21].

The suborder Oncholaimina appears to encompass both the Oxystominidae and the Oncholaimoidea; these families were consistently recovered as a major clade within the Enoplida, in line with Van Megen *et al*.'s [10] topology. Morphological evidence also lends support to this proposed relationship-Lorenzen noted that species from these two families possess 'orthometaneme' type metanemes with associated caudal filaments [18]; most other Enoplid taxa that possess this specific type of metaneme do not have associated caudal filaments, with the exception of a few isolated species. Despite a large number of specimens included from the Oncholaimoidea/Oxystominidae, the internal topology of subclades in this lineage remains largely unresolved.

The present data firmly support early historical classifications [11,15,16] and previous molecular studies [10,33,34] which placed the Trefusiidae within the Enoplida. Terrestrial genera *Trischistoma *and *Tripylina *were also recovered within the Enoplida, despite their historical placement within the Dorylaimia based on morphology [16,18] or within the Triplonchida according to molecular evidence [8,21,23]. Holterman *et al*. [9] first suggested the placement of *Trischistoma *within the Enoplida; Meldal [35] confirmed that both *Trischistoma *and *Tripylina *were excluded from the Triplonchida, but was unable to resolve their exact placement. Our analysis here (like that of Van Megen *et al*. [10]) strongly indicated that the Trefusiidae includes, or is closely associated with, the genera *Trischistoma *and Tripylina. This investigation consistently recovered the Tripyloididae as a sister taxon to the clade containing the Trefusiidae, *Tripylina*, and *Trischistoma*. Such a relationship was first suggested by Siddiqi [19] who placed the Tripylidae species and the Tripyloididae together in the order Tripylida based on morphology. The molecular framework outlined by Van Megen et al. [10] recovered a similar association between these taxa, although this relationship demonstrated low support values in their analysis (ML bootstraps <50%).

Within the Tripyloididae, morphological evidence seems to support the observed separation between *Bathylaimus *and *Tripyloides *in 28S phylogenies (Additional file 2, Figure S2). Taxonomic descriptions outline distinct anatomical differences between these two genera, relating to buccal cavity morphology, amphid shape, shape and position of cervical setae, tail shape, and spicule shape in male specimens [20]. The extent of this morphological differentiation seems to suggest an older split between the two genera (as suggested by 28S topologies) rather than a more recent divergence (e.g. according to 18S data).

Ribosomal topologies suggest that the Ironidae and Alaimina may reside within a single suborder; these two groups were usually recovered as sister taxa, and this relationship was highly supported in Bayesian topologies (0.97). The exact placement of the Ironidae/Alaimina clade was not fully resolved; some trees denoted this clade as an independent lineage within the Enoplida, while other topologies suggested a sister relationship with the Tripyloididae/Trefusiidae clade (e.g. Figure 1a). Previous molecular investigations returned similar results [7,9,10]; molecular data confirmed the placement of the Alaimina and Ironidae within the Enoplida, but other studies were also unable to resolve the exact placement of these taxa. Historical morphological classifications have also struggled to firmly place these two groups. The Ironidae and Alaimina were formerly classed within either the Dorylaimia [11,15,17,18,36] or the Enoplida [13,16]. Several taxonomists proposed a close relationship between the Ironidae and the Tripylidae [13-15], and both Filipjev [11] and Chitwood and Chitwood [13] insinuated a close relationship between the Ironidae and Alaimina. Only a handful of gene sequences from each group were included in the present analysis; increased sampling in future molecular studies may be able to firmly resolve the placement of these two groups.

This study supports previous topologies [10] that denote the Rhabdolaimidae, the genus *Syringolaimus *and *Campydora demonstrans *as members of an independent, highly supported clade. However, our analyses do not support a sister relationship between *Syringolaimus *and *Campydora *that was previously recovered by Van Megen *et al*. [10]. The overall placement of this divergent clade amongst Enoplid lineages was not well resolved. Morphological evidence has previously suggested a close relationship between the Rhabdolaimidae and *Syringolaimus*; most classifications grouped *Syringolaimus *within the Ironidae [11,14,15,18], but some placed this genus within the Rhabdolaimidae in the Araeolaimida [16,37]. The genus *Campydora *was historically classified within the Dorylaimia based on morphology [15,36,38]; only Siddiqi [19] was the only author to suggest a relationship with the Enoplia based on the structure of the pharynx and amphids. The current placement of *Syringolaimus *clearly denotes the polyphyly of the Ironidae *sensu *Lorenzen [18], a finding that was originally put forth by Meldal *et al*. [7] upheld in later phylogenies [10]. Our analyses suggest that the Syringolaimus/*Campydora*/Rhabdolaimidae clade represents a sister group to the Tripyloididae/Trefusiidae clade; however, support values for this sister relationship were usually very low in ML topologies (<50%), and not significantly high in Bayesian phylogenies (0.91).

The placement of the Anticomidae suggests that species within this group represent a major Enoplid family. Previous morphological classifications largely considered the Anticomidae as a subfamily within the Leptsomatidae [11,14-16] and only a few authors separated this group and raised it to family rank [18,39]. Lorenzen's [18] separation of the Anticomidae was based on the left-hand position of gonads and the existence of pre-anal tubules in males of these species (the latter feature not being present in any members of the Leptosomatidae). Although the Leptsomatidae and Anticomidae are both placed within the Enoploidea, these taxa were never recovered as a monophyletic clade, supporting the morphological separation of these two groups.

The Anoplostomatidae *sensu *Lorenzen [18] appears to be an artificial, polyphyletic taxon. The two member genera, *Anoplostoma *and *Chaetonema*, were originally grouped together on the basis of buccal cavity and cephalic capsule structure; however, this study never recovered these two genera within a monophyletic group, despite both being consistently recovered within the superfamily Enoploidea. Previous morphological classifications placed *Chaetonema *within the Enchelidiidae [11] or within the Enoploidea as a member of the Rhabdodemaniidae [15,16]. *Anoplostoma *was first placed within the Phanodermatidae [11], but later excluded completely from the Enoploidea and considered part of the Oncholaimoidea [11,14-16]. Most historical taxonomic classifications did not suggest a close relationship between *Chaetonema *and *Anoplostoma*, and phylogenetic analysis confirms that these two genera represent independent lineages.

Molecular data upholds the classification of the Thoracostmopsidae, Enoplidae, and Phanodermatidae defined by Lorenzen [18]. These three taxa form a large, well-supported clade within the Enoploidea, supporting previous morphological classifications of this superfamily [14-18]. Tree topologies also suggest a sister relationship between the Phanodermatidae and *Enoplus *(the sole genus within the Enoplidae); support values for this pairing were weak in most trees, despite this topology being consistently recovered across different datasets and phylogenetic parameters. Morphological similarities between Thoracostomopsidae and Phanodermatidae were previously suggested by Lorenzen [18], supporting the association between these two groups in molecular frameworks. Lorenzen markedly diverged from earlier classifications by considering *Enoplus *as the only genus within the Enoplidae, basing this separation on differences in metaneme structure, gland arrangements, and the absence of onchia. All other genera previously placed within this group were moved into the Thoracostomopsidae (divided into subfamilies Thoracostomopsinae, Trileptiinae, and Enoplolaiminae). Our investigation only included Thoracostomopsidae species representing the subfamily Enoplolaiminae; further analysis is needed to determine whether genera within the subfamilies Thoracostomopsinae and Trileptiinae also belong within the Thoracostomopsidae *sensu *Lorenzen.

Cryptic diversity has recently been uncovered in well-known marine nematode species [40,41], and evidence from the Enoplida has revealed further unexpected genetic diversity in several groups. Ribosomal 18S and 28S data suggest that the genera *Oncholaimus *and *Viscosia *(Oncholaimoidea) represent artificial, paraphyletic taxonomic groupings. Both groups are known to be taxonomically difficult genera, containing many species with very similar morphology. For specimens identified during the present study, it was often impossible to definitively assign an Oncholaimid specimen as belonging to either genus; the position of the largest subventral tooth was usually the only character that could be used to identify the correct genus of female specimens, and this feature was often obscured if a specimen was awkwardly mounted or exhibited a buccal cavity filled with detritus. Ribosomal topologies imply that morphologically similar *Oncholaimus *and *Viscosia *species represent multiple divergent lineages within the Oncholaimoidea (Additional file 2, Figure S1), suggesting that both genera should be the focus of an extensive taxonomic revision. High genetic diversity was also observed within the morphologically homogeneous Phanodermatidae; species within this family have few distinct anatomical characters, possessing a smooth cuticle, typical pocket-shaped amphids, and little buccal cavity ornamentation. Specimens identified during this study did not display any distinct differences in morphological features, despite ribosomal topologies indicating a complex genetic structuring. The Oncholaimoidea and Phanodermatidae represent the two most densely sampled groups within this study-similarly intensive sampling efforts will be needed to determine true extent of molecular diversity in other nematode families.

### Phylogenetic splits according to habitat

Internal relationships within the Enoplida demonstrate that nematode lineages are primarily separated according to habitat (Figure 1A). Both Bayesian and Maximum Likelihood tree topologies resolved two primary Enoplid clades: one comprised entirely of terrestrial/freshwater species (the Triplonchida), and a second clade containing mostly marine taxa and a few freshwater species (the Enoploidea, Alaimina, Tripyloididae, etc.). The phylogenetic structure of the Enoplida suggests that habitat transitions have occurred at least five times during the evolution of the Enoplida, supporting previous evidence proposing that such habitat transitions are relatively common amongst nematode species [42]. Within the Tripyloididae/Trefusiidae clade, the terrestrial genera *Trischistoma *and *Tripylina *may have arisen from marine ancestors, with the Trefusiidae representing a subsequent reversal back to marine habitats. The genus *Campydora *and the Rhabdolaimidae are two terrestrial taxa that also appear to have arisen independently in a divergent nematode lineage. The phylogenetic relationships in Figure 1A also suggest that marine species of the Ironidae (e.g. *Dolicholaimus sp*.) may be descendents of terrestrial ancestors, although further taxon sampling within the Ironidae may be needed to clearly elucidate patterns within this clade. Past morphological classifications did not typically separate Enoplid genera according to habitat-Maggenti [43] was the sole taxonomist to propose separate terrestrial and marine lineages within the Enoplia (superorders Marenoplica and Terraenoplica), whereas other authors vociferously dismissed this structure [19]. Molecular data appears to support a division primarily (but not exclusively) based on habitat, with several transitions scattered throughout the tree. Citing multiple habitat switches within the Chromadorida, Holterman *et al*. [42] suggested that nematode species only need simple physiological adaptations to move between different physical environments, supporting this seemingly arbitrary pattern of transitions observed in the Enoplida.

### Resolving the earliest splits within Nematoda

Despite exhaustive topological tests and greatly improved taxon sampling, our large-scale phylogenies were unable to resolve the earliest-splitting lineage within the Phylum Nematoda. Dense taxon sampling has greatly improved recent molecular frameworks [10], but our results indicate that the placement of certain clades is unstable even using a large-scale sampling effort. In the this study, Maximum Likelihood methods using a large SSU dataset alternatively recovered both the terrestrial Dorylaimid clade and the primarily marine Enoplid clade as the earliest-splitting nematode group, while large-scale Bayesian analyses consistently returned a polytomy at the basal node of the nematode tree.

There are two primary viewpoints regarding outgroup choice during phylogeny reconstruction. Some authors believe that the most reliable phylogenies are obtained when using the closest sister taxa as an outgroup [44-46], while others advocate the inclusion of both close relatives and more distantly related species [47,48]. An alternative tactic focuses on alignment positions; eliminating divergent (and thus, poorly aligned) sites increases the likelihood of homology (and correct phylogenies) if alignments are unambiguous [49,50]. Although the relationships amongst Metazoan phyla are still hotly debated, mounting molecular evidence supports the Nematomorpha as the sister phylum to the Nematoda [31,51-53]. Taking into account different phylogenetic viewpoints, it would be reasonable to argue that the most robust clade placements should occur when using the Nematomorpha alone as an outgroup (representing the closest relative), multiple non-nematode phyla in combination, or utilising Gblocks-trimmed alignments containing only well-aligned sites. However, these three scenarios give conflicting results: the Dorylaimia is always recovered as the earliest-splitting clade when the Nematomorpha is used as a single outgroup (Figure 2), while the Enoplia is most often observed as the earliest splitting lineage when multiple non-nematode phyla are included in gene alignments. When variable ribosomal regions are removed from alignments using the Gblocks program, the Dorylaimia instead splits off first when multiple non-nematode phyla are present.

Many authors currently assume that Enoplids are earliest branching nematode group [9,23] and both Holterman *et al*. [9] and Van Megen *et al*. [10] reported the Enoplida as the earliest splitting lineage in their published phylogenies, although support values for this topology were low (0.81 PP and 63% ML bootstrap, respectively). In addition, neither study details any rigorous empirical tests to confirm the stability of tree topologies. Molecular frameworks of nematodes generally agree that the Enoplia branched off at some early point in evolutionary history of the phylum [7,9,10,54]-while it is certainly possible that this group diverged first from other nematode groups, the current evidence to support this hypothesis is not strong. We find highest bootstrap support instead for an alternative scenario, the terrestrial Dorylaimia being part of a basal split. Our tests however were unable to resolve either topology as significantly more likely than the other and we do not consider this topology yet resolved.

This unstable topology observed amongst early-splitting clades is likely related to the choice of a single, conserved gene for phylogeny reconstruction. Currently, the 18S gene is the only locus known to resolve deep phylogenetic relationships amongst nematodes. Other genes such as LSU and *cox1 *are only informative at shallower taxonomic levels [29]; in this study, neither gene produced coherent tree topologies for inferring deeper clade relationships (Additional file 2, Figures S2 and S3). Resolving the base of the nematode tree will require intensive efforts to locate other informative genes-ideally protein-coding-which can supplement evolutionary inferences from SSU data. Phylogeny reconstruction in other taxa has already embraced multi-gene phylogenies [e.g. [55,56]], and efforts are now moving towards phylogenomic methods [57,58].

## Conclusions

The sampling effort in this study was by no means exhaustive; isolated specimens represent only a small portion of the diversity within the Enoplida and many genera still remain unsampled in this ubiquitous marine group. Nevertheless, increased taxon sampling within the order Enoplida was able to clearly elucidate major clades and clarify evolutionary relationships amongst genera. The internal structure of the Enoplida is consistent and supported by different analysis methods and data from multiple genetic loci; the resulting molecular phylogenies exhibit clear differences between past morphological classifications and have further refined the molecular framework first proposed by De Ley and Blaxter [8]. Tree topologies suggest that the two main clades within the Enoplia are primarily separated according to habitat (consisting of the terrestrial Triplonchida and mostly marine Enoplida), with habitat transitions occurring at least five times amongst Enoplid species. Ribosomal sequence data further suggests that some morphologically homogenous groups (e.g. Oncholaimidae, Phanodermatidae) exhibit extensive molecular diversity, and further investigation will be required to fully describe this unexpected genetic structure and subsequently revise taxonomic frameworks. Despite dense taxon sampling and rigorous empirical tests, our large-scale phylogenies were unable to recover a well-supported topology amongst early-splitting lineages. The Dorylaimia and Enoplia were both recovered as the earliest-branching clade using a wide range of phylogenetic parameters and outgroup taxa. Future molecular studies of nematodes will need to incorporate phylogenomic methods in order to resolve longstanding questions regarding relationships at the base of the nematode tree.

## Methods

### Materials

Samples were collected from several intertidal locations (coastal sites in New England, the United Kingdom, and South Africa), as well as offshore sediments (off-coast California, Bellinghausen Sea, Southern Indian Ocean, and Iberian Margin). All marine sediments were immediately fixed in DESS preservative [59] using an equal ratio of preservative to sediment. The meiofauna fraction of all samples was extracted *via *decantation and floatation in Ludox^® ^using a 45 *μ*m sieve according to the methods of Somerfield *et al*. [60]. Individual nematodes were picked out of the meiofauna fraction using a fine wire instrument, mounted on slides, and identified down to genus level; video capture images were recorded for all specimens in order to retain a voucher of morphology before specimens were destroyed for molecular analysis.

### Isolation and sequencing of 18S rRNA genes

Genetic data was obtained from a total of 254 Enoplid nematodes, with both 18S rRNA (Accession numbers HM564399-HM564654) and 28S rRNA (Accession numbers HM564655-HM564910) obtained from every specimen; mitochondrial *cox1 *sequences were additionally obtained from a subset of 99 specimens (Accession numbers HM564911-HM565012). Genomic DNA was extracted using a proteinase K digestion [9]. Individual specimens were picked into microcentrifuge tubes containing 25 *μ*l distilled water, followed by the addition of 25 *μ*l lysis buffer (containing 0.2 M NaCl, 0.2 M Tris-HCl (pH 8.0), 1% *β*-mercaptoethanol and 800 *μ*g/ml proteinase K). Tubes were incubated for 2 h at 65°C and 750 rpm in an Eppendorf Thermomixer (Eppendorf, Hamburg, Germany), followed by a final 5 min at 100°C and 750 rpm. Final lysates were stored at −20°C. All PCR reactions were conducted using a DyNAzyme EXT PCR kit (New England Biolabs, Ipswich, MA, USA), with a final reaction volume of 25.75 *μ*l. Each reaction contained 2*μ*l of nematode genomic DNA, 18.25 *μ*l sterile water, 0.4 *μ*M of each primer (Integrated DNA technologies, Coralville, IA, USA) 2.5 *μ*l 10× DyNAzyme EXT Buffer containing MgCl2 (final reaction volume 1.5 mM MgCl2), 0.5 *μ*l dNTP mix containing 10 *μ*M each nucleotide, and 0.5 *μ*l DyNAzyme EXT DNA polymerase (0.5 enzyme units in final reaction volume). Nearly full-length 18S rRNA genes (~1600 bp) were amplified from all nematodes using primer sets G18S4 and 26R, 22F and 13R, and 24F1 and 18P [7,22]. The D2/D3 expansion segment of the 28S rRNA gene (~600 bp) was additionally amplified from all specimens using primers D2A and D3B [28]. A fragment of the *cox1 *gene (~400 bp) was isolated from a subset of 99 nematodes using primers JB3 and JB5 [30]. The following PCR profile was used to amplify all primer sets: 94°C for 5 min followed by 35 cycles of denaturation at 94°C for 30 seconds, annealing at 54°C for 45 seconds, extension at 72°C for 2 minutes, with a final extension of 72°C for 10 min. All PCR products were visualized on a 1.5% agarose gel containing ethidium bromide.

Successful PCR reactions were purified using a QIAquick PCR purification kit (QIAGEN, Valencia, CA, USA). Sequencing reactions were carried out using a BigDye Terminator v3.1 cycle sequencing kit (Applied Biosystems, Foster City, CA, USA), with individual sequencing reactions having a final volume of 10 *μ*l. Each reaction contained 3 *μ*l 5X ABI sequencing buffer, 2 *μ*l of 2 *μ*M forward or reverse primer, 1 *μ*l BigDye Terminator v1.1, and either 2*μ*l or 4 *μ*l of purified PCR product. Sequencing reactions were carried out using the following thermal profile: 96°C for 1 minute followed by 25 cycles of 96°C for 10 seconds, 50°C for 5 seconds, and 60°C for 4 minutes. Cycle-sequence products were purified via ethanol precipitation and sequenced using an ABI 3130 genetic analyzer.

### Sequence Alignment and Phylogenetic Analysis

Pre-aligned structural alignments of the 18S rRNA gene were downloaded from release 98 of the SILVA rRNA database [61] and imported into the ARB software suite [62]. Enoplid sequences generated during this investigation were incorporated into nematode secondary structure alignments via the Positional Tree (PT) Server function in the ARB software suite. Alignment quality was assessed by first constructing Neighbour-Joining trees in ARB; some manual editing was necessary to ensure that all secondary structure motifs were properly aligned. Short sequences (<1000 bp) and sequences of dubious quality were removed from the alignment. The Tardigrada, Kinorhyncha, Priapulida and Nematomorpha were chosen as outgroups, representing the closest relatives of the Phylum Nematoda [31]; final alignments contained up to 1428 sequences, incorporating nematode and outgroup taxa (final ARB databases containing 18S and 28S alignments are available in Additional file 3). In total, the 18S dataset contained 354 unique sequences (including 52 nematodes from the Triplonchida) representing 37 genera from the order Enoplida. In this study, LSU sequences (~600 bp representing the D2/D3 expansion segment) were obtained from every Enoplid nematode represented by an 18S sequence; amplification difficulties meant that *cox1 *(~400 bp) was only obtained from a subset of 94 specimens in total. In addition, fewer LSU and *cox1 *sequences were available from GenBank. Final LSU datasets contained 280 Enoplid sequences, and final *cox1 *datasets contained 105 taxa.

Structural alignments were used to construct large-scale Maximum Likelihood trees using Randomized Axelerated Maximum Likelihood (RAxML) version 7.04 [63,64], hosted at the Vital-IT unit of the Swiss Institute of Bioinformatics (http://phylobench.vital-it.ch/raxml-bb/). Support values were generated from RAxML runs using 100 bootstrap replicates. Bayesian inference was used to supplement topological inferences. Data was submitted to the CIPRES project cluster hosted at the University of California, San Diego and analysed using MrBayes3.2 (http://www.phylo.org/sub_sections/portal/); datasets were run for up to 4,000,000 generations using the GTR+I+G model of nucleotide substitution, 4 MCMC chains, and a heating temperature of 0.06.

Deep phylogeny was investigated using the 18S rRNA gene, using both a large dataset (representing all major nematode clades), as well as a smaller dataset comprising only Enoplid and Dorylaimid taxa. Small subunit phylogenies were built using both Maximum Likelihood and Bayesian Inference methods (Figure 1). Large-scale Maximum Likelihood phylogenies were constructed using 1336 nematode taxa and utilised four closely related metazoan phyla [31] as outgroup taxa (Nematomorpha, Priapulida, Kinorhyncha, and Tardigrada). The small dataset contained a total of 377 Enoplid sequences and utilised Dorylaimid nematodes as outgroup taxa. Extensive tests were carried out on Maximum Likelihood phylogenies in order to determine the robustness of tree topologies; trees were constructed using different phylogenetic parameters and combinations of taxa. The stability of clade placements was assessed in comparison with published phylogenies and other tree topologies obtained during the present study. The placement of major nematode clades and the internal topology of the Enoplida were evaluated in every phylogeny. Nematode taxa were analysed alongside both single and multiple outgroups, using different outgroup combinations to assess topological changes. Secondary structure information was used to separate gene alignments according to stem and loop structures present in folded ribosomal subunits; tree topologies from these partitioned gene alignments were compared to non-partitioned ML runs. To assess the impact of seemingly rogue taxa, long-branch clades and taxa of *incertae sedis *were both removed and included in analyses to test for any potentially destabilising effects. Phylogenies were constructed using outputs from the Gblocks program [50], which selects conserved blocks from 18S alignments and eliminates poorly aligned sites and potentially saturated or overly divergent regions. Finally, trees were constructed both with and without the P-invar parameter in RAxML which estimates the proportion of invariable sites, as there is some evidence to suggest that this parameter interferes with estimates of among-site rate variation [65-67].

Both 28S and *cox1 *sequences were used to supplement inferences from 18S data and provide further resolution at lower taxonomic levels. Despite downloading pre-aligned LSU structural alignments from the online SILVA database, the LSU dataset was poorly aligned and it was difficult to infer homology amongst variable regions. However, it was still possible to align sequences within closely related taxa (below the family level) and construct Maximum Likelihood trees. LSU datasets contained a total of 393 taxa, utilising Dorylaimid sequences for outgroup comparisons. Protein-coding *cox1 *sequences were analysed using both Maximum Likelihood and Bayesian Inference, with gene alignments partitioned according to codon position. Datasets contained a total of 105 taxa, with sequences from *Pellioditis marina *(a Rhabditid nematode) used for outgroup comparisons. One mitochondrial sequence (TCR 89, *Litinium sp*.) exhibited a very long branch length within both ML and Bayesian topologies; additional trees were constructed without this particular sequence, but excluding this long-branch taxon did not have any impact on tree topology.

The support for alternate topologies was assessed using the Shimodaira-Hasegawa log likelihood test [32] implemented in RAxML. Nematomorpha were used as outgroup and alternate topologies varied the groups involved in the basal nematode split.

## Authors' contributions

HMB carried out nematode extraction and sequencing, sequence alignment, tree building, and drafted the manuscript. DHL participated in sequence alignment and tree building. WKT participated in nematode sequencing and aided in designing the study. PJDL conceived the study and participated in its design and coordination. All authors read and approved the final manuscript.

## Supplementary Material

Additional file 1 **List of all taxa included in phylogenetic analysis**. List of all sequences utilised during this study, including taxonomic identification, accession number, and genetic locus. Click here for file

Additional file 2 **Supplementary 18S, 28S and cox1 phylogenies**. Figure S1: Expanded Maximum Likelihood 18S phylogeny of the Enoplia, fully expanded and annotated with taxonomic classifications. Tree built using 18S sequence data from 81 taxa, with estimation of the P-Invar parameter and partitions according to 18S secondary structure. The black square within Oncholaimoidea represent the primary shallow-water clades containing the majority of *Oncholaimus *and *Viscosia *specimens isolated in this study. Figure S2: Maximum Likelihood phylogeny of the nematode subclass Enoplia, built using sequences from the D2/D3 expansion region of the 28S gene. Tree constructed using 433 taxa, with estimation of P-Invar parameter. No alignment regions were excluded from the analysis. Figure S3: Bayesian phylogeny of the nematode order Enoplida built using *cox1 *gene sequences. Tree constructed using a 3 alignment partitions according to codon positions, using 2 million generations, and chain heating temperature of 0.1. Click here for file

Additional file 3 **ARB databases containing 18S and 28S structural alignments for all nematode taxa used in phylogenetic analyses**. Final rRNA gene alignments utilised for phylogenetic analyses in this study, contained in a database accessible using the ARB software suite (http://www.arb-home.de/). Click here for file
