# Supplementary material for: Moving towards a complete molecular framework of the Nematoda: a focus on the Enoplida and early-branching clades
Source: BMC Evol Biol. 2010 Nov 12;10:353. doi: 10.1186/1471-2148-10-353 (PMC2995457; doi:10.1186/1471-2148-10-353)
Supplement: Additional file 1 — List of all taxa included in phylogenetic analysis. List of all sequences utilised during this study, including taxonomic identification, accession number, and genetic locus. [file 1471-2148-10-353-S1.PDF]

Genbank Accession numbers of 18S, 28S and *cox1* sequences amplified during this investigation. Parentheses denote uncertain genus assignments.

Seq. ID	Taxonomic ID	SSU	LSU	Cox1
AUK 1	*Tripyloides sp.*			HM564911
AUK 7	*Tripyloides sp.*			HM564915
AUK 10	*Viscosia sp.*	HM564399	HM564655	
AUK 13	*Calyptronema sp.*	HM564400	HM564656	HM564912
AUK 14	*Oxystomina sp.*	HM564401	HM564657	
AUK 18	*Calyptronema sp.*			HM564913
AUK 23	*Oncholaimus sp.*	HM564402	HM564658	HM564914
AUK 35	*Oncholaimus sp.*	HM564474	HM564730	
AUK 36	*Oncholaimus sp.*	HM564475	HM564731	
AUK 45	*Tripyloides sp.*	HM564476	HM564732	
BAUK 9	*Oxystomina sp.*	HM564403	HM564659	
BCA 1	*Syringolaimus sp.*	HM564477	HM564733	
BCA 2	*Syringolaimus sp.*	HM564485	HM564741	
BCA 3	*Pareurystomina*	HM564491	HM564746	
BCA 5	*Syringolaimus sp.*	HM564500	HM564755	
BCA 6	*Syringolaimus sp.*	HM564501	HM564756	
BCA 10	*Trefusia sp.*	HM564478	HM564734	
BCA 12	*Halalaimus sp.*	HM564479	HM564735	
BCA 14	*Mesacanthion/Paramesacanthion sp.*	HM564480	HM564736	
BCA 15	*Oxystomina sp.*	HM564481	HM564737	
BCA 16	*Halalaimus sp.*	HM564482	HM564738	
BCA 17	*Halalaimus sp.*	HM564483	HM564739	
BCA 19	*Mesacanthion/Paramesacanthion sp.*	HM564484	HM564740	
BCA 20	Phanodermatidae sp.	HM564486	HM564742	
BCA 21	*Oxystomina sp.*	HM564487	HM564881	
BCA 22	*Oxystomina sp.*	HM564488	HM564743	
BCA 23	*Oxystomina sp.*	HM564489	HM564744	
BCA 25	*Halalaimus sp.*	HM564490	HM564745	HM564934
BCA 26	*Oncholaimus sp.*			HM564935
BCA 31	*Syringolaimus sp.*	HM564492	HM564747	
BCA 32	Phanodermatidae sp.	HM564493	HM564748	
BCA 35	*Oxystomina sp.*	HM564494	HM564749	
BCA 37	Phanodermatidae sp.	HM564495	HM564750	HM564936
BCA 38	*Halalaimus sp.*	HM564496	HM564751	
BCA 40	*Bathyeurystomina sp.*			HM564937
BCA 41	*Syringolaimus sp.*	HM564497	HM564752	
BCA 42	*Oxystomina sp.*	HM564498	HM564753	HM564938
BCA 47	*Syringolaimus sp.*	HM564499	HM564754	

BUS 1	*Oncholaimus sp.*	HM564404	HM564660	HM564916
BUS 2	*Oncholaimus sp.*	HM564406	HM564662	HM564917
BUS 3	*Oncholaimus sp.*	HM564408	HM564664	HM564918
BUS 4	*Oncholaimus sp.*	HM564409	HM564665	HM564919
BUS 5	*Oncholaimus sp.*	HM564410	HM564666	HM564920
BUS 7	*Oncholaimus sp.*	HM564411	HM564667	HM564921
BUS 15	*Tripyloides sp.*	HM564405	HM564661	
BUS 21	*Anoplostoma sp.*	HM564407	HM564663	
Cr 1	Thoracostomopsidae *sp.*	HM564412	HM564669	HM564922
Cr 3	*Phanodermopsis sp.*	HM564413	HM564668	HM564923
Cr 4	*Halalaimus sp.*			HM564924
Cr 7	*Halalaimus sp.*	HM564414	HM564687	
Cr 9	*Halalaimus sp.*	HM564415	HM564688	
Cr 11	*Halalaimus sp.*	HM564502	HM564810	
Cr 13	*Halalaimus sp.*	HM564503	HM564811	
Cr 18 b	*Mesacanthion/ Paramesacanthion sp.*	HM564504	HM564812	
Cr 19 b	*Phanodermopsis sp.*	HM564505	HM564813	
Cr 20 b	*Halalaimus sp.*	HM564506	HM564814	
Cr 21 b	Comesomatidae	HM564507	HM564815	
Cr 24 b	*Metaparoncholaimus/Meyersia sp.*	HM564508	HM564908	HM564939
Cr 26	Phanodermatidae sp.	HM564509	HM564816	
Cr 33	*Phanodermopsis sp.*	HM564510	HM564817	
Cr 34	*Mesacanthion/ Paramesacanthion sp.*	HM564511	HM564818	
Cr 35	*Halalaimus sp.*	HM564512	HM564819	
Cr 38	Anticomidae sp.	HM564513	HM564820	
Cr 54	*Phanodermopsis sp.*	HM564514	HM564821	
Cr 55	*Halalaimus sp.*	HM564515	HM564822	
Cr 56	*Phanodermopsis sp.*	HM564516	HM564823	
Cr 59	*Halalaimus sp.*	HM564517	HM564824	HM564940
Cr 60	*Halalaimus sp.*	HM564518	HM564825	
Cr 61	*Halalaimus sp.*	HM564519	HM564826	
Cr 62	*Halalaimus sp.*	HM564520	HM564827	
Cr 63	*Halalaimus sp.*	HM564521	HM564882	
Cr 64	*Halalaimus sp.*	HM564522	HM564883	
Cr 66	*Phanodermopsis sp.*	HM564523	HM564884	HM564941
Cr 68	*Phanodermopsis sp.*	HM564524	HM564885	HM564942
Cr 72a	*Halalaimus sp.*	HM564526	HM564887	
Cr 73a	*Chaetonema sp.*	HM564528	HM564828	
Cr 74a	*Halalaimus sp.*	HM564530	HM564890	
Cr 76a	*Chaetonema sp.*	HM564533	HM564893	
Cr 77a	*Oxystomina sp.*	HM564535	HM564895	
Cr 78a	*Bathyeurystomina sp.*	HM564537	HM564897	

Cr 80a	*Oxystomina sp.*	HM564538	HM564898	
Cr 82a	*Halalaimus sp.*	HM564540	HM564900	
Cr 83a	*Chaetonema sp.*	HM564542	HM564901	
Cr 85a	*Halalaimus sp.*	HM564545	HM564904	
Cr 71b	*Phanodermopsis sp.*	HM564525	HM564886	
Cr 72b	Phanodermatidae sp.	HM564527	HM564888	HM564943
Cr 73b	*Halalaimus sp.*	HM564529	HM564889	
Cr 74b	*Halalaimus sp.*	HM564531	HM564891	
Cr 75b	*Halalaimus sp.*	HM564532	HM564892	
Cr 76b	*Oxystomina sp.*	HM564534	HM564894	
Cr 77b	*Halalaimus sp.*	HM564536	HM564896	
Cr 80b	*Bathyeurystomina sp.*	HM564539	HM564899	
Cr 82b	Thoracostomopsidae sp.	HM564541	HM564909	HM564944
Cr 83b	*Halalaimus sp.*	HM564543	HM564902	
Cr 84b	*Chaetonema sp.*	HM564544	HM564903	
Cr 85b	*Halalaimus sp.*	HM564546	HM564905	
Cr 86	*Halalaimus sp.*	HM564547	HM564906	
Cr 87	*Oxystomina sp.*	HM564548	HM564907	
DBA 1	*Enoploides sp.*	HM564549	HM564757	HM564945
DBA 2	*Enoploides sp.*	HM564550	HM564758	HM564946
DBA 3	*Enoploides sp.*	HM564552	HM564760	
DBA 4	*Oncholaimus sp.*	HM564553	HM564761	HM564947
DBA 5	*Enoploides sp.*	HM564554	HM564762	HM564948
DBA 6	*Enoploides sp.*	HM564555	HM564764	HM564949
DBA 7	*Enoploides sp.*	HM564556	HM564763	HM564950
DBA 21	*Enoplus sp.*	HM564551	HM564759	
HCL 2	Oncholaimidae sp.	HM564561	HM564769	
HCL 5	Oncholaimidae sp.	HM564568	HM564776	
HCL 7	Oncholaimidae sp.	HM564569	HM564777	
HCL 8	Oncholaimidae sp.			HM564952
HCL 9	*Viscosia sp.*	HM564570	HM564778	
HCL 10	*Viscosia sp.*	HM564557	HM564765	
HCL 11	*Viscosia sp.*	HM564558	HM564766	
HCL 12	Oncholaimidae sp.	HM564559	HM564767	
HCL 15	*Viscosia sp.*	HM564560	HM564768	
HCL 20	*Oxystomina sp.*	HM564562	HM564770	
HCL 21	*Oxystomina sp.*	HM564563	HM564771	
HCL 23	Oncholaimidae sp.	HM564564	HM564772	HM564951
HCL 24	*Viscosia sp.*	HM564565	HM564773	
HCL 27	*Viscosia sp.*	HM564566	HM564774	
HCL 32	*Oxystomina sp.*	HM564567	HM564775	
HUK 1	Oncholaimidae sp.	HM564416	HM564689	HM564985

JCC 4	*Anticoma sp.*			HM564954
JCC 23	Phanodermatidae sp.	HM564571	HM564779	
JCC 29	Anticomidae *sp.*	HM564572	HM564829	
JCC 37	*Enoplolaimus sp.*			HM564953
JCC 52	Phanodermatidae sp.	HM564573	HM564780	HM564955
JCC 59	*Phanodermopsis sp.*	HM564574	HM564781	HM564956
JCC 79	*Anticoma sp.*			HM564957
JCC 89	*Phanodermopsis sp.*	HM564575	HM564782	
LCL 1	*Trefusia sp.*	HM564576	HM564783	
LCL 2	*Trefusia sp.*	HM564578	HM564785	
LCL 3	*Trefusia sp.*	HM564581	HM564788	HM564960
LCL 4	*Trefusia sp.*	HM564582	HM564789	
LCL 5	*Bathylaimus sp.*	HM564583	HM564790	HM564961
LCL 7	*Trefusia sp.*	HM564584	HM564791	HM564962
LCL 8	*Trefusia sp.*	HM564585	HM564792	
LCL 9	*Bathylaimus sp.*	HM564586	HM564793	HM564963
LCL 19	*Bathylaimus sp.*	HM564577	HM564784	HM564958
LCL 20	Oncholaimidae sp. (Viscosia sp.)	HM564579	HM564786	
LCL 21	*Bathylaimus sp.*	HM564580	HM564787	HM564959
LUK 1	*Viscosia sp.*	HM564417	HM564670	
LUK 3	*Viscosia sp.*	HM564419	HM564672	
LUK 6	*Halalaimus sp.*	HM564420	HM564673	
LUK 7	*Calyptronema sp.*	HM564421	HM564674	HM564926
LUK 12	*Calyptronema sp.*	HM564418	HM564671	HM564925
NAR 1	*Enoplolaimus sp.*	HM564422	HM564690	HM564986
NAR 2	*Enoplolaimus sp.*	HM564427	HM564695	HM564990
NAR 4	*Oncholaimus sp.*	HM564429	HM564697	
NAR 5	*Enoplolaimus sp.*	HM564430	HM564698	HM564991
NAR 6	*Chaetonema sp.*	HM564431	HM564699	HM564992
NAR 7	*Oncholaimus sp.*	HM564432	HM564700	HM564993
NAR 8	*Enoplolaimus sp.*	HM564433	HM564701	HM564994
NAR 9	*Enoplolaimus sp.*	HM564434	HM564702	HM564995
NAR 11	*Bathylaimus sp.*	HM564423	HM564691	HM564987
NAR 14	*Bathylaimus sp.*	HM564424	HM564692	HM564988
NAR 15	*Bathylaimus sp.*	HM564425	HM564693	
NAR 16	*Oncholaimus sp.*	HM564426	HM564694	HM564989
NAR 20	*Bathylaimus sp.*	HM564428	HM564696	
NUS 1	*Pareurystomina sp.*	HM564435	HM564675	
NUS 2	*Oncholaimus sp.*	HM564438	HM564678	HM564928
NUS 3	*Oxystomina sp.*	HM564440	HM564680	
NUS 4	*Oncholaimus sp.*	HM564441	HM564681	HM564930

NUS 5	*Oncholaimus sp.*	HM564444	HM564684	HM564931
NUS 6	*Oncholaimus sp.*	HM564445	HM564685	HM564932
NUS 7	*Oncholaimus sp.*	HM564446	HM564686	HM564933
NUS 10	*Oncholaimus sp.*			HM564927
NUS 11	*Bathylaimus sp.*	HM564436	HM564676	
NUS 14	*Tripyloides sp.*	HM564437	HM564677	
NUS 21	*Oxystomina sp.*	HM564439	HM564679	HM564929
NUS 40	*Anoplostoma*	HM564442	HM564682	
NUS 41	*Tripyloides sp.*	HM564443	HM564683	
OUS 1	Oncholaimidae sp.	HM564447	HM564703	HM564996
OUS 2	*Oncholaimus sp.*	HM564450	HM564706	HM564998
OUS 3	*Anoplostoma sp.*	HM564453	HM564709	HM564999
OUS 4	*Halalaimus sp.*	HM564454	HM564710	
OUS 5	*Anoplostoma sp.*	HM564455	HM564711	HM565000
OUS 6	*Anoplostoma sp.*	HM564456	HM564712	HM565001
OUS 7	*Anoplostoma sp.*	HM564457	HM564713	
OUS 8	*Anoplostoma sp.*	HM564458	HM564714	
OUS 9	Oncholaimidae sp.	HM564459	HM564715	
OUS 10	*Enoploides sp.*	HM564448	HM564704	
OUS 14	Oncholaimidae sp.	HM564449	HM564705	HM564997
OUS 21	Oncholaimidae sp.	HM564451	HM564707	
OUS 22	*Halalaimus sp.*	HM564452	HM564708	
PPA 1	*Enoplolaimus sp.*			HM564964
PPA 3	*Enoplolaimus sp.*			HM564965
PPA 5	*Enoplolaimus sp.*			HM564966
PPA 7	*Enoplus sp.*	HM564587	HM564794	HM564967
SBA 1	*Halalaimus sp.*	HM564588	HM564795	
SBA 2	*Oncholaimus sp.*	HM564592	HM564799	HM564970
SBA 3	*Oncholaimus sp.*	HM564593	HM564800	HM564971
SBA 5	*Oncholaimus sp.*	HM564594	HM564801	HM564972
SBA 7	Thoracostomopsidae sp.			HM564973
SBA 8	Thoracostomopsidae sp.			HM564974
SBA 9	Thoracostomopsidae sp.			HM564975
SBA 10	*Halalaimus sp.*	HM564589	HM564796	
SBA 12	*Halalaimus sp.*	HM564590	HM564797	
SBA 13	Thoracostomopsidae sp.	HM564591	HM564798	HM564968
SBA 14	Thoracostomopsidae sp.			HM564969
SBN 2	*Viscosia sp.*	HM564595	HM564802	
SBN 3	*Oxystomina sp.*	HM564596	HM564803	
SBN 4	*Viscosia sp.*	HM564597	HM564804	
SUS 1	*Enoplolaimus sp.*	HM564460	HM564716	HM565002

SUS 2	*Enoplolaimus sp.*	HM564463	HM564719	HM565005
SUS 6	*Enoplolaimus sp.*	HM564466	HM564722	HM565007
SUS 10	*Enoplolaimus sp.*	HM564461	HM564717	HM565003
SUS 15	*Enoplolaimus sp.*	HM564462	HM564718	HM565004
SUS 21	*Enoplolaimus sp.*	HM564464	HM564720	HM565006
SUS 27	Oncholaimidae sp.	HM564465	HM564721	
TCR 1	*Halalaimus sp.*	HM564598	HM564830	
TCR 3	*Halalaimus sp.*	HM564636	HM564809	
TCR 12	Oncholaimidae sp.	HM564605	HM564805	
TCR 13	*Halalaimus sp.*	HM564608	HM564838	
TCR 17	Oncholaimidae sp.	HM564620	HM564806	
TCR 21	*Oxystomina sp.*	HM564631	HM564807	
TCR 26	*Halalaimus sp.*	HM564635	HM564808	
TCR 41	*(Bathyeurystomina sp.)*			HM564977
TCR 42	*Oncholaimidae sp.*	HM564637	HM564862	
TCR 44	*Anticoma sp.*	HM564638	HM564863	
TCR 68	*Oxystomina sp.*	HM564639	HM564864	
TCR 69	Oncholaimidae sp.	HM564640	HM564865	
TCR 70	Phanodermatidae sp.	HM564641	HM564866	HM564978
TCR 74	Thoracostomopsidae sp.	HM564642	HM564867	
TCR 75	Phanodermatidae sp.	HM564643	HM564868	HM564979
TCR 78	*Phanodermopsis sp.*	HM564644	HM564869	HM564980
TCR 80	*Phanodermopsis sp.*	HM564645	HM564870	
TCR 81	*Bathyeurystomina sp.*	HM564646	HM564871	HM564981
TCR 82	Comesomatidae sp.	HM564647	HM564872	
TCR 87	*Bathylaimus sp.*	HM564648	HM564873	HM564982
TCR 89	*Litinium sp.*	HM564649	HM564874	HM564983
TCR 90	*Litinium sp.*	HM564650	HM564875	
TCR 91	*Oxystomina sp.*	HM564651	HM564876	
TCR 93	*Halalaimus sp.*	HM564652	HM564877	
TCR 94	*Mesacanthion/ Paramesacanthi*on sp.	HM564653	HM564878	
TCR 95	*Enoploides sp.*			HM564984
TCR 97	*Cricohalalaimus sp.*	HM564654	HM564879	
TCR 102	Thoracostomopsidae sp.	HM564599	HM564831	
TCR 106	*Bathyeurystomina sp.*	HM564600	HM564832	
TCR 108	*Phanodermopsis sp.*	HM564601	HM564833	
TCR 109	*Bathyeurystomina sp.*	HM564602	HM564834	
TCR 112	*Halalaimus sp.*	HM564603	HM564835	
TCR 114	*Dolicholaimus sp.*	HM564604	HM564836	
TCR 125	*Rhabdocoma sp.*	HM564606	HM564837	
TCR 128	*Bathyeurystomina sp.*	HM564607	HM564910	
TCR 130	*Rhabdocoma sp.*	HM564609	HM564839	

TCR 131	*Halalaimus sp.*	HM564610	HM564840	
TCR 139	*Rhabdocoma sp.*	HM564611	HM564841	
TCR 141	*(Cephalanticoma sp.)*	HM564612	HM564842	
TCR 143	*Enoplolaimus/Mesacanthion sp.*	HM564613	HM564843	
TCR 145	*Syringolaimus sp.*	HM564614	HM564844	
TCR 148	*Phanodermopsis sp.*	HM564615	HM564845	
TCR 149	Anticomidae sp.	HM564616	HM564846	
TCR 152	*Phanodermopsis sp.*	HM564617	HM564847	
TCR 153	Phanodermatidae sp.	HM564618	HM564848	
TCR 158	*Mesacanthion/Paramesacanthion sp.*	HM564619	HM564849	
TCR 173	Phanodermatidae sp.	HM564621	HM564850	
TCR 180	*Oxystomina sp.*	HM564622	HM564851	
TCR 184	*(Epicanthion sp.)*	HM564623	HM564852	
TCR 188	*(Phanodermopsis sp.)*	HM564624	HM564853	
TCR 190	*Phanodermopsis sp.*	HM564625	HM564854	
TCR 192	*Leptosomatides sp.*	HM564626	HM564855	
TCR 197	*Anticoma sp.*	HM564627	HM564856	HM564976
TCR 202	*Oxystomina sp.*	HM564628	HM564857	
TCR 205	*Litinium sp.*	HM564629	HM564858	
TCR 206	*Synonchus sp.*	HM564630	HM564859	
TCR 212	*(Oxystomina sp.)*	HM564632	HM564860	
TCR 216	*(Phanodermopsis sp.)*	HM564633	HM564861	
TCR 230	*Thalassoalaimus sp.*	HM564634	HM564880	
WUS 1	*Enoplolaimus sp.*	HM564467	HM564723	HM565008
WUS 2	*Enoplolaimus sp.*	HM564468	HM564724	HM565009
WUS 3	*Enoplolaimus sp./Mesacanthion sp.*	HM564469	HM564725	
WUS 4	*Enoplolaimus sp.*	HM564470	HM564726	HM565010
WUS 5	*Enoplolaimus sp.*	HM564471	HM564727	HM565011
WUS 6	*Enoplolaimus sp./Mesacanthion sp.*	HM564472	HM564728	
WUS 7	*Enoplolaimus sp.*	HM564473	HM564729	HM565012

Published gene sequences included in phylogenetic analyses:

Genetic Locus	Taxonomic ID (from EMBL)	Accession No.
18S	Acanthocheilonema viteae	DQ094171
18S	Acanthopharynx micans	Y16911
18S	Achromadora cf terricola JH-2004	AY593940
18S	Achromadora ruricola	AY593941
18S	Achromadora sp. JH-2004	AY284718
18S	Achromadora sp. JH-2004	AY284717
18S	Acrobeles ciliatus	AF202148
18S	Acrobeles complexus	AY284671
18S	Acrobeles maximus	EU196016

18S	Acrobeles sp.	U81576
18S	Acrobeloides apiculatus	AY284673
18S	Acrobeloides bodenheimeri	AF202162
18S	Acrobeloides bodenheimeri	AF202159
18S	Acrobeloides buetschlii	EU543174
18S	Acrobeloides maximus	EU306344
18S	Acrobeloides nanus	AY284672
18S	Acrobeloides nanus	DQ102707
18S	Acrobeloides sp. PS1146	AF034391
18S	Acrobeloides thornei	EU543175
18S	Adoncholaimus fuscus	AY854195
18S	Adoncholaimus sp.	AF036642
18S	Aduncospiculum halicti	U61759
18S	Aelurostrongylus abstrusus	AJ920366
18S	Alaimus parvus	AY284738
18S	Alaimus sp. 1247	FJ040489
18S	Alaimus sp. PDL-2005	AJ966514
18S	Alinema amazonicum	DQ442672
18S	Allodorylaimus andrassyi	AY284801
18S	Allodorylaimus sp. PDL-2005	AJ966472
18S	Amidostomum cygni	AJ920353
18S	Amplimerlinius icarus	EU306351
18S	Anaplectus grandepapillatus	AY284697
18S	Anaplectus grandepapillatus	AY284698
18S	Anaplectus porosus	FJ040453
18S	Anaplectus porosus	AY284696
18S	Anaplectus sp. PDL-2005	AJ966473
18S	Anatonchus tridentatus	AJ966474
18S	Ancylostoma caninum (dog hookworm)	AJ920347
18S	Ancylostoma duodenale	EU344798
18S	Angiostrongylus cantonensis	AY295804
18S	Angiostrongylus costaricensis	DQ116748
18S	Angiostrongylus costaricensis	EF514913
18S	Angiostrongylus dujardini	EF514915
18S	Angiostrongylus malaysiensis	EF514914
18S	Angiostrongylus vasorum	EF514916
18S	Angiostrongylus vasorum	AJ920365
18S	Anguillicola crassus	DQ490223
18S	Anguillicola crassus	DQ118535
18S	Anguina tritici	AY593913
18S	Anisakis pegreffi	EF180082

18S	Anisakis sp.	U94365
18S	Anisakis sp.	U81575
18S	Anomyctus xenurus	FJ040413
18S	Anoplostoma rectospiculum	AY590149
18S	Anoplostoma sp. 1058	FJ040491
18S	Anoplostoma sp. 1093	FJ040492
18S	Anoplostoma sp. BHMM-2005	AY854194
18S	Anoplostoma sp. PB-2005	AM235215
18S	Aphanolaimus aquaticus	AY593932
18S	Aphanolaimus aquaticus	AY593933
18S	Aphanonchus cf. europaeus AphNEurZ1	EF591319
18S	Aphelenchoides besseyi	AY508035
18S	Aphelenchoides bicaudatus	AY284643
18S	Aphelenchoides blastophtorus	AY284644
18S	Aphelenchoides cf. bicaudatus MHMH-2008	FJ040407
18S	Aphelenchoides fragariae	AB067755
18S	Aphelenchoides fragariae	AY284645
18S	Aphelenchoides fragariae	AJ966475
18S	Aphelenchoides ritzemabosi	DQ901554
18S	Aphelenchoides sp. 2130	FJ040410
18S	Aphelenchoides sp. 2137	FJ040412
18S	Aphelenchoides sp. JB011	DQ901550
18S	Aphelenchoides sp. JB012	DQ901553
18S	Aphelenchoides sp. JH-2004	AY284646
18S	Aphelenchoides sp. JH-2004	AY284647
18S	Aphelenchoides sp. SAS-2006	DQ901552
18S	Aphelenchoides stammeri	AB368535
18S	Aphelenchus avenae	AY284640
18S	Aphelenchus avenae	AY284639
18S	Aphelenchus avenae	AF036586
18S	Aphelenchus avenae	EU306347
18S	Aphelenchus avenae	AB368918
18S	Aphelenchus sp. JH-2004	AY284641
18S	Aporcelaimellus cf. paraobtusicaudatus JH-2004	AY284812
18S	Aporcelaimellus obtusicaudatus	AY284811
18S	Aporcelaimellus obtusicaudatus	DQ141212
18S	Aporcelaimellus sp. F2	AJ875153
18S	Aporcelaimellus sp. G2	AJ875154
18S	Aquatides christei	AY552963
18S	Ascaridia galli	EF180058
18S	Ascaris lumbricoides (common roundworm)	U94366

18S	Ascaris suum (pig roundworm)	U94367
18S	Ascaris suum (pig roundworm)	AF036587
18S	Ascarophis arctica	DQ094172
18S	Ascolaimus cf. elongatus AscoElo2Z	EF591330
18S	Ascolaimus cf. elongatus MHMH-2008	FJ040460
18S	Ascolaimus elongatus	AY854231
18S	Ascolaimus elongatus	AM234617
18S	Aspiculuris tetraptera	EF464551
18S	Aspidodera sp. SAN-2007	EF180070
18S	Astomonema sp. NCM-2006	DQ408760
18S	Astomonema sp. NCM-2006	DQ408759
18S	Astomonema sp. NCM-2006	DQ408761
18S	Aulolaimus oxycephalus	AY284724
18S	Axonchium propinquum	AY284820
18S	Axonolaimus helgolandicus	AY854232
18S	Axonolaimus sp. 1088	FJ040461
18S	Axonolaimus sp. 1277	FJ040462
18S	Axonolaimus sp. AxLaSp2	EF591331
18S	Basiria gracilis	EU130839
18S	Bastiania gracilis	AY284726
18S	Bastiania gracilis	AY284725
18S	Bathylaimus assimilis	AJ966476
18S	Bathylaimus sp. 1263	FJ040504
18S	Bathylaimus sp. BHMM-2005	AY854201
18S	Bathylaimus sp. PB-2005	AM234619
18S	Bathyodontus cylindricus	AY552964
18S	Bathyodontus mirus	AY284744
18S	Baujardia mirabilis	AF547385
18S	Baylisascaris procyonis	U94368
18S	Baylisascaris transfuga	U94369
18S	Belonolaimus longicaudatus	AY633449
18S	Belonolaimus longicaudatus	EU130838
18S	Bitylenchus dubius	AY284601
18S	Boleodorus thylactus	AY593915
18S	Boleodorus thylactus	AY993976
18S	Bradynema listronotum	DQ915805
18S	Brevibucca saprophaga	EU196018
18S	Brevibucca sp. SB261	AF202163
18S	Brugia malayi	AAQA01004941
18S	Brugia malayi	AAQA01003643
18S	Brugia malayi	AAQA01004529

18S	Brugia malayi	AAQA01004416
18S	Brugia malayi	AAQA01005177
18S	Brugia malayi	AAQA01004588
18S	Brugia malayi	AAQA01010496
18S	Brugia malayi	AAQA01009726
18S	Brugia malayi	AF036588
18S	Brugia malayi	AAQA01003418
18S	Brumptaemilius justini	AF036589
18S	Bunonema franzi	AJ966477
18S	Bunonema reticulatum	AY284661
18S	Bunonema reticulatum	FJ040450
18S	Bunonema reticulatum	AY593925
18S	Bunonema reticulatum	EU196017
18S	Bunonema richtersi	FJ040452
18S	Bunonema richtersi	FJ040451
18S	Bunonema sp.	U81582
18S	Bursaphelenchus abruptus	AY508010
18S	Bursaphelenchus borealis	AY508012
18S	Bursaphelenchus cocophilus (red ring nematode)	AY509153
18S	Bursaphelenchus fraudulentus	AB067758
18S	Bursaphelenchus hylobianum	AY508019
18S	Bursaphelenchus poligraphi	AY508028
18S	Bursaphelenchus sexdentati	AY508031
18S	Caenorhabditis brenneri	U13930
18S	Caenorhabditis briggsae	U13929
18S	Caenorhabditis briggsae	FJ380929
18S	Caenorhabditis drosophilae	AF083025
18S	Caenorhabditis elegans	EU196001
18S	Caenorhabditis elegans	Z92784
18S	Caenorhabditis elegans	Z92784
18S	Caenorhabditis japonica	AY602182
18S	Caenorhabditis plicata	AY602178
18S	Caenorhabditis remanei	U13931
18S	Caenorhabditis sp. DF5070	AY602181
18S	Caenorhabditis sp. JU727	EU196000
18S	Californidorus sp. Arkansas-WY-2003	AY283155
18S	Calomicrolaimus parahonestus	AY854218
18S	Calyptronema maxweberi	AY854199
18S	Calyptronema sp. 1068	FJ040503
18S	Camacolaimus sp. CamaSp1	EF591325
18S	Camacolaimus sp. CamaSp2	EF591327

18S	Camallanus cotti	EF180071
18S	Camallanus cotti	DQ442662
18S	Camallanus lacustris	DQ442663
18S	Camallanus oxycephalus	DQ503463
18S	Camallanus sp. MW-2006	DQ442664
18S	Campydora demonstrans	AY552965
18S	Carcharodiscus banaticus	AY284827
18S	Catanema sp.	Y16912
18S	Cephalenchus hexalineatus	AY284594
18S	Cephalobidae sp. MHMH-2008	FJ040406
18S	Cephaloboides cf. armata SB363	EU196005
18S	Cephaloboides nidrosiensis	EU196020
18S	Cephaloboides sp. SB227	AF083027
18S	Cephalobus cubaensis	AF202161
18S	Cephalobus oryzae	AF034390
18S	Cephalobus persegnis	AY284662
18S	Cephalobus persegnis	AY284663
18S	Cephalobus sp. PS1143	AF202158
18S	Cephalobus sp. PS1196	AF202160
18S	Ceratoplectus armatus	AY284706
18S	Cervidellus alutus	AF202152
18S	Cervidellus sp. JH-2004	AY284674
18S	cf. Tylencholaimus sp. JH-2004	AY284833
18S	cf. Tylencholaimus sp. JH-2004	AY284832
18S	Chabertia ovina	AJ920341
18S	Chiloplacus propinquus	AY284677
18S	Choanolaimus psammophilus	FJ040467
18S	Choanolaimus psammophilus	AY284716
18S	Choriorhabditis cristata	EU196013
18S	Choriorhabditis dudichi	AF083012
18S	Chromadora nudicapitata	AY854205
18S	Chromadora sp. BHMM-2005	AY854206
18S	Chromadorid sp. JH-2004	AY284713
18S	Chromadoridae sp. MHMH-2008	FJ040474
18S	Chromadorina germanica	AY854207
18S	Chromadorina sp. 1257	FJ040470
18S	Chromadorina sp. 1971	FJ040471
18S	Chromadorita cf. leuckarti MHMH-2008	FJ040473
18S	Chromadorita tentabundum	AY854208
18S	Chromadoropsis vivipara	AF047891
18S	Chronogaster boettgeri	AY593931

18S	Chronogaster sp. 1189	FJ040455
18S	Chronogaster sp. JH-2004	AY284709
18S	Chronogaster sp. JH-2004	AY284708
18S	Chronogaster typica	FJ040456
18S	Chrysonema attenuatum	AY284779
18S	Chrysonema attenuatum	EF207245
18S	Chrysonema attenuatum	AY593945
18S	Clarkus papillatus	AY552966
18S	Clarkus papillatus	AY284750
18S	Clarkus papillatus	AY284749
18S	Clarkus papillatus	AY284748
18S	Clarkus sp. PDL-2005	AJ966479
18S	Clavicaudoides sp. PGM-2004	AY552967
18S	Clavicaudoides trophurus	AY593943
18S	Clavicaudoides trophurus	AY284772
18S	Clavicaudoides trophurus	AY284773
18S	Contracaecum eudyptulae	EF180072
18S	Contracaecum microcephalum	AY702702
18S	Contracaecum multipapillatum	U94370
18S	Coomansus parvus	AY284766
18S	Coomansus parvus	AY284767
18S	Coslenchus cf. franklinae	AY284582
18S	Coslenchus costatus	AY284581
18S	Coslenchus franklinae	AY284583
18S	Crenosoma mephitidis	AY295805
18S	Crenosoma vulpis	AJ920367
18S	Criconema sp. PDL-2005	AJ966480
18S	Crustorhabditis scanica	AF083014
18S	Cruzia americana	U94371
18S	Cruznema sp. JH-2004	AY284658
18S	Cruznema sp. JH-2004	AY284657
18S	Cruznema sp. JH-2004	AY284656
18S	Cruznema sp. JH-2004	AY284655
18S	Cruznema tripartitum	EU196012
18S	Cruznema tripartitum	U73449
18S	Cryptonchus sp. 1453	FJ040479
18S	Cryptonchus tristis	EF207244
18S	Cuticonema vivipara	EU196019
18S	Cuticularia sp.	U81583
18S	Cuticularia sp. PS-2006	DQ385848
18S	Cyartonema elegans	AY854203
18S	Cyatholaimidae sp. BHMM-2005	AY854212

18S	Cyatholaimus sp. BHMM-2005	AY854213
18S	Cyatholaimus sp. PB-2005	AM234618
18S	Cyclodontostomum purvisi	AJ920340
18S	Cylicocyclus insignis	AJ920342
18S	Cylindrolaimus communis	AY593939
18S	Cylindrolaimus sp. 202149	AF202149
18S	Cyrnea leptoptera	EU004815
18S	Cyrnea mansioni	AY702701
18S	Cyrnea seurati	EU004816
18S	Daptonema hirsutum	AY854223
18S	Daptonema normandicum	AY854224
18S	Daptonema oxycerca	AY854225
18S	Daptonema procerus	AF047889
18S	Daptonema setosum	AM234045
18S	Daptonema sp. 1255	FJ040463
18S	Daptonema sp. PFN-2007	EF436228
18S	Deladenus siricidicola	FJ004890
18S	Deladenus siricidicola	EU545475
18S	Deladenus siricidicola	AY633447
18S	Deladenus sp. 1 WB-2008	EU306345
18S	Deletrocephalus dimidiatus	AJ920346
18S	Demaniella sp. 2007	FJ040438
18S	Dentiphilometra sp. MW-2006	DQ442673
18S	Dentostomella sp.	AF036590
18S	Deontolaimus papillatus	EF591322
18S	Deontolaimus papillatus	FJ040457
18S	Desmodora communis	AY854215
18S	Desmodora ovigera	Y16913
18S	Desmolaimus sp. DeLaSp1	EF591332
18S	Desmolaimus sp. DeLaSp2	EF591333
18S	Desmolaimus zeelandicus	AY854229
18S	Desmoscolex sp. DeCoSp2	EF591342
18S	Dichromadora sp. 1260	FJ040506
18S	Dichromadora sp. BHMM-2005	AY854209
18S	Dictyocaulus capreolus	AY168862
18S	Dictyocaulus eckerti	AY168857
18S	Dictyocaulus filaria	AJ920362
18S	Dictyocaulus filaria	AY168861
18S	Dictyocaulus sp. P6A1	AY168860
18S	Dictyocaulus viviparus (bovine lungworm)	AJ920361
18S	Dictyocaulus viviparus (bovine lungworm)	AY168856

18S	Didelphostrongylus hayesi	AY295806
18S	Dintheria tenuissima	FJ040487
18S	Dipetalonema sp. YQ-2006	DQ531723
18S	Diphterophora communis	AY593955
18S	Diphterophora obesa	AY284839
18S	Diphterophora obesa	AY284838
18S	Diphtherophora obesus	AY552968
18S	Diplogasterid sp. JH-2004	AY284689
18S	Diplogasteroides magnus	FJ040448
18S	Diplolaimella dievengatensis	AJ966482
18S	Diplolaimelloides meyli	AF036611
18S	Diplolaimelloides meyli	AF036644
18S	Diplolaimelloides sp. BCG-2008	EU551671
18S	Diplopeltula sp. DiPeSp1	EF591329
18S	Diploscapter coronatus	AY593921
18S	Diploscapter sp.	U81586
18S	Diploscapter sp. JU359	EU196003
18S	Diploscapter sp. PS1897	AF083009
18S	Dirofilaria immitis (dog heartworm nematode)	AF036638
18S	Dirofilaria immitis (dog heartworm nematode)	AF182647
18S	Discolaimus cf. major HHBM-2007a	EF207252
18S	Discolaimus major	AY284828
18S	Distolabrellus veechi	AF082999
18S	Distolabrellus veechi	AF083011
18S	Ditylenchus adasi	EU669909
18S	Ditylenchus angustus	AJ966483
18S	Ditylenchus brevicauda	AY284635
18S	Ditylenchus destructor	EU188752
18S	Ditylenchus destructor	EU188745
18S	Ditylenchus destructor	EU188729
18S	Ditylenchus destructor	EU188748
18S	Ditylenchus destructor	AY593912
18S	Ditylenchus destructor	EU188744
18S	Ditylenchus dipsaci	AY284636
18S	Ditylenchus dipsaci	AY593911
18S	Ditylenchus dipsaci	EU669931
18S	Ditylenchus dipsaci	AY593906
18S	Ditylenchus dipsaci	AY593909
18S	Ditylenchus dipsaci	AY593908
18S	Ditylenchus sp. 1 JH-2003	AY284637
18S	Ditylenchus sp. WY-2004	AY589297

18S	Dolichodorus sp. WY-2006	DQ912918
18S	Domorganus macronephriticus	FJ040454
18S	Dorylaimellus montenegricus	AY284821
18S	Dorylaimellus virginianus	AY552969
18S	Dorylaimoides limnophilus	AY593950
18S	Dorylaimoides micoletzkyi	AY284830
18S	Dorylaimoides sp. JH-2004	AY593951
18S	Dorylaimopsis punctata	AM234047
18S	Dorylaimus stagnalis	AY284776
18S	Dorylaimus stagnalis	AY284777
18S	Dracunculus insignis	AY947719
18S	Dracunculus medinensis	AY947720
18S	Dracunculus medinensis	AY852268
18S	Dracunculus oesophageus	AY852269
18S	Dracunculus sp. V3104	DQ503457
18S	Drilocephalobus sp. JH-2004	AY284678
18S	Drilocephalobus sp. JH-2004	AY284679
18S	Dujardinascaris waltoni	EF180081
18S	Echinuria borealis	EF180064
18S	Ecphyadophora sp. JH-2004	AY593917
18S	Ecphyadophora tenuissima	EU669911
18S	Ecphyadophora tenuissima	EU669910
18S	Ecumenicus monohystera	AY284783
18S	Ecumenicus monohystera	AY284784
18S	Ecumenicus sp. JH-2004	AY284781
18S	Ecumenicus sp. JH-2004	AY284782
18S	Ektaphelenchus obtusus	AB368532
18S	Enchodelus macrodorus	AY284791
18S	Enchodelus sp. HHBM-2007a	EF207247
18S	Enchodelus sp. JH-2004	AY284792
18S	Enchodelus sp. JH-2004	AY284793
18S	Enoploides brunettii	AY854193
18S	Enoploides sp. 1252	FJ040490
18S	Enoploides sp. PB-2005	AM234621
18S	Enoplus brevis	U88336
18S	Enoplus communis	AY854192
18S	Enoplus meridionalis	Y16914
18S	Epidorylaimus lugdunensis	AY284803
18S	Epidorylaimus lugdunensis	AY284802
18S	Epidorylaimus sp. 1457	FJ040478
18S	Epsilonematidae sp. Epsifamil1	EF591340

18S	Ethmolaimus pratensis	AY593942
18S	Ethmolaimus pratensis	FJ040475
18S	Eubostrichus dianae	Y16915
18S	Eubostrichus parasitiferus	Y16916
18S	Eubostrichus topiarius	Y16917
18S	Eucephalobus cf. oxyuroides JH-2004	AY284664
18S	Eucephalobus oxyuroides	AY284665
18S	Eucephalobus striatus	AY284667
18S	Eucephalobus striatus	AY284666
18S	Eudorylaimus carteri	AJ966484
18S	Eudorylaimus sp. JH-2004	AY284800
18S	Eumonhystera cf. similis JH-2004	AY284691
18S	Eumonhystera cf. simplex JH-2004	AY284692
18S	Eumonhystera filiformis	AY593937
18S	Euteratocephalus palustris	AY284684
18S	Euteratocephalus sp. JH-2004	AY284685
18S	Fergusobia sp. 19	EF029084
18S	Fergusobia sp. 281	AY589298
18S	Fergusobia sp. 282	AY589299
18S	Fergusobia sp. 329	AY589301
18S	Fergusobia sp. 330	AY589302
18S	Fergusobia sp. 339	AY589303
18S	Fergusobia sp. 357	AY633448
18S	Fergusobia sp. 39	AY589292
18S	Fergusobia sp. 421	EF011666
18S	Fergusobia sp. 444	EF011667
18S	Fergusobia sp. 451	EF011669
18S	Fergusobia sp. 465	EF011670
18S	Fergusobia sp. 469	EF011671
18S	Fergusobia sp. 54	AY589294
18S	Fergusobia sp. 56	AY589295
18S	Fictor sp. 2011	FJ040437
18S	Filarinema flagrifer	AJ920354
18S	Filarioid sp. JK-2007	EF081340
18S	Filaroides martis	AY295807
18S	Filenchus filiformis	AY284592
18S	Filenchus thornei	AY284591
18S	Geocenamus quadrifer	AY993977
18S	Geomonhystera sp. 1998	FJ040465
18S	Geomonhystera villosa	EF591334
18S	Globodera achilleae	FJ040399

18S	Globodera artemisiae	EU855121
18S	Globodera artemisiae	FJ040400
18S	Globodera pallida	AY284620
18S	Globodera pallida	AY593875
18S	Globodera pallida	EU855119
18S	Globodera pallida	AF036592
18S	Globodera rostochiensis	AY593881
18S	Globodera rostochiensis	EU855120
18S	Globodera tabacum	FJ040401
18S	Gnathostoma binucleatum	Z96946
18S	Gnathostoma neoprocyonis	Z96947
18S	Gnathostoma turgidum	Z96948
18S	Goezia pelagia	U94372
18S	Granonchulus sp. JH-2004	AY593953
18S	Haemonchus contortus	EU086375
18S	Haemonchus contortus	L04153
18S	Haemonchus contortus	EU086374
18S	Haemonchus sp. V3091	DQ503465
18S	Halalaimus sp. 1034	FJ040501
18S	Halenchus fucicola	EU669912
18S	Halicephalobus gingivalis	AF202156
18S	Halichoanolaimus sp. HaChSp1	EF591338
18S	Haliplectus sp. JH-2004	AY593935
18S	Halocercus invaginatus	AY295808
18S	Halomonhystera disjuncta	AJ966485
18S	Helicotylenchus canadensis	AY284605
18S	Helicotylenchus dihystera	AJ966486
18S	Helicotylenchus pseudorobustus	AY284606
18S	Helicotylenchus varicaudatus	EU306354
18S	Helicotylenchus vulgaris	AY284607
18S	Heligmosomoides polygyrus	AJ920355
18S	Heligmosomoides polygyrus	AY542283
18S	Hemicriconemoides pseudobrachyurus	AY284622
18S	Hemicriconemoides pseudobrachyurus	AY284624
18S	Hemicriconemoides pseudobrachyurus	AY284623
18S	Hemicycliophora conida	EU669914
18S	Hemicycliophora conida	AJ966471
18S	Hemicycliophora thienemanni	EU306341
18S	Herpetostrongylus pythonis	AJ920358
18S	Heterakis gallinarum	DQ503462
18S	Heterakis sp. 14690	AF083003

18S	Heterocephalobus elongatus	AY284669
18S	Heterocephalobus elongatus	AY284668
18S	Heterocephalobus elongatus	AY284670
18S	Heterocheilus tunicatus	U94373
18S	Heterodera avenae	FJ040403
18S	Heterodera betae	FJ040404
18S	Heterodera glycines	ABLA01015961
18S	Heterodera glycines	ABLA01013124
18S	Heterodera glycines	ABLA01017282
18S	Heterodera glycines	ABLA01014545
18S	Heterodera glycines	ABLA01014793
18S	Heterodera glycines	ABLA01016090
18S	Heterodera glycines	ABLA01017129
18S	Heterodera glycines	ABLA01019319
18S	Heterodera glycines	ABLA01020458
18S	Heterodera goettingiana	EU669915
18S	Heterodera hordecalis	FJ040405
18S	Heterodera koreana	EU306357
18S	Heterodera mani	EU669916
18S	Heterodera schachtii	AY284617
18S	Heterodera schachtii	EU306355
18S	Heterodera trifolii	FJ040402
18S	Heterorhabditidoides chongmingensis	EF503692
18S	Heterorhabditis bacteriophora	FJ040429
18S	Heterorhabditis bacteriophora	FJ040430
18S	Heterorhabditis bacteriophora	AF036593
18S	Heterorhabditis bacteriophora	FJ040428
18S	Heterorhabditis hepialus	AF083004
18S	Heterorhabditis marelatus	FJ040431
18S	Heterorhabditis megidis	FJ040433
18S	Heterorhabditis megidis	FJ040434
18S	Heterorhabditis megidis	FJ040432
18S	Heterorhabditis sp. 1395	FJ040435
18S	Heterorhabditis zealandica	AJ920368
18S	Hirschmanniella cf. belli ITDL-2006	EF029856
18S	Hirschmanniella gracilis	EU669959
18S	Hirschmanniella loofi	EU306353
18S	Hirschmanniella pomponiensis	EF029854
18S	Hirschmanniella santarosae	EF029855
18S	Hirschmanniella sp. 1 JH-2003	AY284614
18S	Hirschmanniella sp. 2 JH-2003	AY284615

18S	Hirschmanniella sp. 3 JH-2003	AY284616
18S	Hirschmanniella sp. Yuma	EF029857
18S	Hovorkonema variegatum	AY702705
18S	Howardula aoronymphium	AY589304
18S	Howardula aoronymphium	AF519224
18S	Howardula cf. aoronymphium	AF519225
18S	Howardula neocosmis	AF519226
18S	Howardula sp. SP-A	AF519232
18S	Howardula sp. SP-B	AF519223
18S	Howardula sp. SP-F	AF519222
18S	Howardula sp. SP-MA	AF519233
18S	Howardula sp. SP-PS	AF519231
18S	Hypodontus macropi	AJ920339
18S	Hysterothylacium fortalezae	U94374
18S	Hysterothylacium pelagicum	U94375
18S	Hysterothylacium reliquens	U94376
18S	Iheringascaris inquies	U94377
18S	Ironus dentifurcatus	AJ966487
18S	Ironus longicaudatus	FJ040495
18S	Ironus sp. 1992	FJ040496
18S	Ironus sp. 2-PM-2004	AY552970
18S	Isolaimium sp. 2-PM-2004	AY552971
18S	Kalicephalus cristatus	AJ920349
18S	Koerneria sp. SB110	EU196025
18S	Labiostrongylus bipapillosus	AJ920337
18S	Labronema ferox	AY552972
18S	Labronema vulvapapillatum	AY284807
18S	Laimaphelenchus penardi	AY593918
18S	Laimaphelenchus penardi	EU306346
18S	Laimaphelenchus penardi	AY593919
18S	Laxus cosmopolitus	Y16918
18S	Laxus oneistus	Y16919
18S	Leidynema portentosae	EF180073
18S	Lelenchus leptosoma	AY284584
18S	Leptolaimus sp. 1283	FJ040458
18S	Leptolaimus sp. LeLaSp1	EF591323
18S	Leptolaimus sp. LeLaSp2	EF591324
18S	Leptonchus granulosus	AY284831
18S	Leptonemella sp.	Y16920
18S	Linhomoeidae sp. DeLaSp3Z	EF591336
18S	Linhomoeidae sp. DeLaSp4Z	EF591337

18S	Litomosoides sigmodontis	AF227233
18S	Loa loa	DQ094173
18S	Longidorella sp. 1 JH-2004	AY284789
18S	Longidorella sp. 2 JH-2004	AY284790
18S	Longidorus attenuatus	AY687994
18S	Longidorus biformis	AY283171
18S	Longidorus crassus	AY283158
18S	Longidorus diadecturus	AY283166
18S	Longidorus dunensis	AY284819
18S	Longidorus elongatus	AY687992
18S	Longidorus euonymus	AY687995
18S	Longidorus grandis	AY283165
18S	Longidorus helveticus	EF538759
18S	Longidorus leptocephalus	EU503142
18S	Longidorus litchii	AY687996
18S	Longidorus macrosoma	EF538758
18S	Longidorus paravineacola	AY283157
18S	Longidorus poessneckensis	EF538757
18S	Longidorus sp. Georgia-WY-2003	AY283168
18S	Longidorus uroshis	EF538760
18S	Longidorus vineacola	AY283169
18S	Loofia thienemanni	AY284629
18S	Loofia thienemanni	AY284628
18S	Macrotrophurus arbusticola	AY284595
18S	Macrotrophurus arbusticola	AY284596
18S	Malenchus andrassyi	AY284587
18S	Margolisianum bulbosum	AB185161
18S	Meloidogyne chitwoodi	EU669934
18S	Meloidogyne chitwoodi	AY593885
18S	Meloidogyne ethiopica	AY942630
18S	Meloidogyne fallax	AY593895
18S	Meloidogyne hapla	ABLG01003285
18S	Meloidogyne hapla	ABLG01000304
18S	Meloidogyne ichinohei	EU669954
18S	Meloidogyne incognita (southern root-knot nematode)	CABB01002720
18S	Meloidogyne incognita (southern root-knot nematode)	CABB01000342
18S	Meloidogyne incognita (southern root-knot nematode)	AY268120
18S	Meloidogyne javanica (root-knot nematode)	AY942626
18S	Meloidogyne javanica (root-knot nematode)	EU669938

18S	Meloidogyne javanica (root-knot nematode)	AY268121
18S	Meloidogyne mali	EU669948
18S	Meloidogyne maritima	EU669944
18S	Meloidogyne microtyla	AF442198
18S	Meloidogyne minor	EU669937
18S	Meloidogyne naasi	AY593901
18S	Meloidogyne paranaensis	AY942622
18S	Meloidogyne ulmi	EU669947
18S	Merlinius brevidens	AY284597
18S	Mermis nigrescens	AF036641
18S	Mermithid sp. JH-2004	AY284743
18S	Mermithidae sp. MHMH-2008	FJ040480
18S	Mesocriconema xenoplax	AY284626
18S	Mesocriconema xenoplax	AY284627
18S	Mesocriconema xenoplax	AY284625
18S	Mesodorylaimus aberrans	AY593947
18S	Mesodorylaimus bastiani	AJ966488
18S	Mesodorylaimus centrocercus	EF207248
18S	Mesodorylaimus centrocercus	AY284799
18S	Mesodorylaimus cf. nigritulus AV-2005	AJ966490
18S	Mesodorylaimus japonicus	AJ966489
18S	Mesodorylaimus sp. JH-2004	AY284780
18S	Mesorhabditis anisomorpha	AF083013
18S	Mesorhabditis longespiculosa	EU543178
18S	Mesorhabditis longespiculosa	EU196014
18S	Mesorhabditis miotki	EU543177
18S	Mesorhabditis sp.	U73452
18S	Mesorhabditis sp. JH-2004	AY284660
18S	Mesorhabditis sp. JH-2004	AY593922
18S	Mesorhabditis spiculigera	AF083016
18S	Metachromadora remanei	AM234620
18S	Metachromadora remanei	AY854216
18S	Metachromadora sp.	AF036595
18S	Metachromadora sp. 1089	FJ040469
18S	Metachromadora sp. MAchSp1	EF591339
18S	Metadesmolaimus sp. PDL-2005	AJ966491
18S	Metaporcelaimus simplex	AY593948
18S	Metastrongylus elongatus	AJ920363
18S	Metastrongylus salmi	AY295809
18S	Metateratocephalus crassidens	AY284687
18S	Metateratocephalus crassidens	AY284686

18S	Metateratocephalus crassidens	AY593934
18S	Miconchus cf. fasciatus	AY552973
18S	Microdorylaimus miser	AY284804
18S	Microdorylaimus modestus	AY284806
18S	Microdorylaimus modestus	AY284805
18S	Microdorylaimus sp. PDL-2005	AJ966492
18S	Micropleura australiensis	DQ442678
18S	Microtetrameres cloacitectus	EU004814
18S	Molgolaimus demani	AY854220
18S	Molnaria intestinalis	DQ442668
18S	Monhystera riemanni	AY593938
18S	Mononchoides striatus	AY593924
18S	Mononchus aquaticus	AY284765
18S	Mononchus aquaticus	AY297821
18S	Mononchus aquaticus	AY284764
18S	Mononchus truncatus	AJ966493
18S	Mononchus truncatus	AY284762
18S	Mononchus tunbridgensis	AY593954
18S	Monoposthia costata	AY854221
18S	Monoposthia sp. 1266	FJ040505
18S	Muellerius capillaris	AY295810
18S	Myctolaimus ulmi	EU196024
18S	Mylonchulus arenicolus	AF036596
18S	Mylonchulus brachyuris	AY284754
18S	Mylonchulus brachyuris	AY284753
18S	Mylonchulus rotundicaudatus	AY284751
18S	Mylonchulus sigmaturus	AY284756
18S	Mylonchulus sigmaturus	AY284757
18S	Mylonchulus sigmaturus	AY284755
18S	Mylonchulus sp. F6	AJ875156
18S	Mylonchulus sp. JH-2004	AY284761
18S	Mylonchulus sp. JH-2004	AY284760
18S	Myolaimus sp.	U81585
18S	Nacobbus aberrans	AF442190
18S	Nacobbus aberrans	AJ966494
18S	Nagelus obscurus	EU306350
18S	Nagelus obscurus	AY593904
18S	Nanidorus nanus	FJ040485
18S	Nanidorus nanus	FJ040486
18S	Necator americanus	AJ920348
18S	Necator americanus	AY295811

18S	Nematodirus battus	U01230
18S	Nematodirus battus	AJ920360
18S	Nemhelix bakeri	DQ118537
18S	Neoascarophis macrouri	DQ442660
18S	Neochromadora BHMM-2005	AY854210
18S	Neodolichorhynchus lamelliferus	AY284598
18S	Neodolichorhynchus microphasmis	EU669917
18S	Neopsilenchus magnidens	AY284585
18S	Nicollina cameroni	AJ920357
18S	Nilonema senticosum	DQ442671
18S	Nippostrongylus brasiliensis	AJ920356
18S	Nippostrongylus brasiliensis	AF036597
18S	Nothotylenchus acris	AY593914
18S	Nudora bipapillata	AY854222
18S	Nygolaimus cf. brachyuris JH-2004	AY284771
18S	Nygolaimus cf. brachyuris JH-2004	AY284770
18S	Nygolaimus cf. parvus	AY552974
18S	Odontopharynx longicaudata	FJ040449
18S	Odontophora rectangula	AY854233
18S	Odontophora sp. 1273	FJ040459
18S	Ogma cobbi	EU669918
18S	Ogma menzeli	EU669919
18S	Onchium sp. OChiSp1	EF591328
18S	Onchocerca cervicalis	DQ094174
18S	Onchocercidae sp. WB-2005	DQ103704
18S	Oncholaimidae sp. MHMH-2008	FJ040493
18S	Oncholaimidae sp. XS-2005	AY866479
18S	Oncholaimus sp. BHMM-2005	AY854196
18S	Opisthodorylaimus sylphoides	AY284785
18S	Oscheius dolichura	EU196010
18S	Oscheius dolichuroides	AF082998
18S	Oscheius guentheri	EU196022
18S	Oscheius insectivora	AF083019
18S	Oscheius sp. BW282	AF082994
18S	Oscheius tipulae	EU196009
18S	Oscheius tipulae	AF036591
18S	Oslerus osleri	AY295812
18S	Ostertagia leptospicularis	AJ920351
18S	Ostertagia ostertagi	AJ920352
18S	Ostertagia ostertagi	AF036598
18S	Otostrongylus circumlitus	AY295813

18S	Otostrongylus sp.	U81589
18S	Ottolenchus discrepans	AY284590
18S	Oxydirus nethus	EF207251
18S	Oxydirus oxycephaloides	AY284823
18S	Oxydirus oxycephalus	AY284825
18S	Oxydirus oxycephalus	AY284824
18S	Oxystomina sp. 1262	FJ040498
18S	Oxystomina sp. 1282	FJ040499
18S	Oxyuris equi	EF180062
18S	Panagrellus redivivus	AF036599
18S	Panagrellus redivivus	AF083007
18S	Panagrobelus stammeri	AF202153
18S	panagrolaimoid nematode KR3021	U81580
18S	Panagrolaimus cf. rigidus AF40	DQ285636
18S	Panagrolaimus davidi	AJ567385
18S	Panagrolaimus detritophagus	EU543176
18S	Panagrolaimus paetzoldi	FJ040414
18S	Panagrolaimus sp. PS1159	U81579
18S	Panagrolaimus subelongatus	AY284681
18S	Paracanthonchus caecus	AF047888
18S	Paractinolaimus macrolaimus	AY993978
18S	Paractinolaimus macrolaimus	AY284826
18S	Paractinolaimus sp. PM-2002	AY552975
18S	Paracyatholaimus intermedius	AJ966495
18S	Parafilaroides decorus	AY295814
18S	Parafilaroides sp.	U81590
18S	Paramphidelus hortensis	AY284739
18S	Paramphidelus sp. JH-2004	AY284742
18S	Paramphidelus sp. JH-2004	AY284741
18S	Paramphidelus sp. JH-2004	AY284740
18S	Paraphelenchus sp. JH-2004	AY284642
18S	Paraplectonema pedunculatum	EF591320
18S	Parascaris equorum	U94378
18S	Parasitorhabditis obtusa	EU003189
18S	Parasitorhabditis sp. SB281	AF083028
18S	Paraspidodera sp. 21303	AF083005
18S	Parastrongyloides trichosuri	AJ417024
18S	Paratrichodorus allius	AJ439572
18S	Paratrichodorus allius	AM087124
18S	Paratrichodorus anemones	AF036600
18S	Paratrichodorus divergens	DQ345528

18S	Paratrichodorus hispanus	AJ439577
18S	Paratrichodorus hispanus	DQ345527
18S	Paratrichodorus macrostylus	AJ439507
18S	Paratrichodorus macrostylus	AJ439622
18S	Paratrichodorus minor	AM269897
18S	Paratrichodorus pachydermus	AJ439574
18S	Paratrichodorus porosus	DQ345524
18S	Paratrichodorus teres	AM087125
18S	Paratripyla sp. JH-2004	AY284737
18S	Paratylenchus cf. neoamblicephalus	AY284634
18S	Paratylenchus dianthus	AJ966496
18S	Paratylenchus microdorus	AY284632
18S	Paratylenchus microdorus	AY284633
18S	Paratylenchus straeleni	AY284630
18S	Paratylenchus straeleni	AY284631
18S	Paravulvus hartingii	AY552976
18S	Paravulvus hartingii	AY284774
18S	Paravulvus hartingii	AY284775
18S	Paraxonchium laetificans	AY284808
18S	Paraxonchium laetificans	AY284809
18S	Paraxonchium laetificans	AY284810
18S	Parelaphostrongylus odocoilei	AY295815
18S	Passalurus ambiguus	EF464552
18S	Passalurus sp. SAN-2007	EF180061
18S	Pellioditis marina	AF083021
18S	Pellioditis mediterranea	AF083020
18S	Pellioditis sp. JU274	EU196011
18S	Pellioditis typica	U13933
18S	Pelodera pseudoteres	EU196023
18S	Pelodera teres	AF083002
18S	Petrovinema poculatum	AJ920343
18S	Phasmarhabditis hermaphrodita	DQ639981
18S	Phasmarhabditis hermaphrodita	DQ639980
18S	Phasmarhabditis sp. EM434	EU196008
18S	Philometra cyprinirutili	DQ442675
18S	Philometra lateolabracis	FJ161972
18S	Philometra madai	FJ161974
18S	Philometra nemipteri	FJ161975
18S	Philometra obturans	AY852267
18S	Philometra ovata	DQ442677
18S	Philometra sciaenae	FJ161971

18S	Philometra sp. 1 KMAQ-2008	FJ161973
18S	Philometra sp. MW-2006	DQ442674
18S	Philometroides sanguineus	DQ442676
18S	Philometroides seriolae	FJ155811
18S	Philonema oncorhynchi	DQ442670
18S	Philonema sp.	U81574
18S	Physaloptera alata	AY702703
18S	Physaloptera apivori	EU004817
18S	Physaloptera sp. SAN-2007	EF180065
18S	Physaloptera turgida	DQ503459
18S	Plectidae sp. PDL-2005	AJ966478
18S	Plectidae sp. PDL-2005	AJ966508
18S	Plectonchus sp. JH-2004	AY593920
18S	Plectonchus sp. PDL0025	AF202154
18S	Plectus acuminatus	AF037628
18S	Plectus aquatilis	AF036602
18S	Plectus aquatilis	AY284700
18S	Plectus cf. cirratus JH-2004	AY284701
18S	Plectus cf. parietinus JH-2004	AY284703
18S	Plectus cf. parietinus JH-2004	AY284702
18S	Plectus cf. parvus JH-2004	AY284699
18S	Plectus rhizophilus	AY593929
18S	Plectus rhizophilus	AY593928
18S	Plectus sp.	U61761
18S	Poikilolaimus oxycercus	AF083023
18S	Poikilolaimus oxycercus	FJ040436
18S	Poikilolaimus regenfussi	AF083022
18S	Poikilolaimus sp. RGD617	AB370214
18S	Pontonema vulgare	AF047890
18S	Porrocaecum angusticolle	EU004820
18S	Porrocaecum depressum	U94379
18S	Porrocaecum streperae	EF180074
18S	Praeacanthonchus punctatus	AY854214
18S	Praeacanthonchus sp.	AF036612
18S	Praeacanthonchus sp. 178510	AM234046
18S	Pratylenchoides magnicauda	AF202157
18S	Pratylenchoides ritteri	AJ966497
18S	Pratylenchus convallariae	EU669957
18S	Pratylenchus crenatus	EU669920
18S	Pratylenchus crenatus	EU669922
18S	Pratylenchus goodeyi	AJ966498

18S	Pratylenchus neglectus	EU669923
18S	Pratylenchus neglectus	EU669924
18S	Pratylenchus penetrans	EU669925
18S	Pratylenchus penetrans	EU669926
18S	Pratylenchus scribneri	EU669958
18S	Pratylenchus scribneri	EU669927
18S	Pratylenchus thornei	EU669928
18S	Pratylenchus thornei	EU669929
18S	Pratylenchus vulnus	EU669956
18S	Pratylenchus vulnus	EU669955
18S	Prionchulus muscorum	AJ966500
18S	Prionchulus muscorum	AY284745
18S	Prionchulus punctatus	AY284746
18S	Prionchulus punctatus	AY284747
18S	Prismatolaimus cf. dolichurus JH-2004	AY284728
18S	Prismatolaimus cf. dolichurus JH-2004	AY284727
18S	Prismatolaimus dolichurus	AY593957
18S	Prismatolaimus intermedius	AY284729
18S	Prismatolaimus intermedius	AF036603
18S	Pristionchus aerivorus	FJ040440
18S	Pristionchus americanus	FJ040445
18S	Pristionchus entomophagus	FJ040441
18S	Pristionchus lheritieri	AY593923
18S	Pristionchus lheritieri	AY284690
18S	Pristionchus lheritieri	AF036640
18S	Pristionchus lheritieri	FJ040439
18S	Pristionchus marianneae	FJ040442
18S	Pristionchus maupasi	FJ040443
18S	Pristionchus pacificus	AF083010
18S	Pristionchus pacificus	U81584
18S	Pristionchus pauli	FJ040446
18S	Pristionchus pseudaerivorus	FJ040447
18S	Pristionchus uniformis	FJ040444
18S	Procamacolaimus sp. PrCoSp1	EF591326
18S	Procamallanus pacificus	DQ442665
18S	Procamallanus pintoi	DQ442666
18S	Procamallanus rebecae	DQ442667
18S	Procephalobus sp. 1 WB-2008	EU543179
18S	Prochromadora sp. ProcSp1	EF591341
18S	Prodesmodora circulata	AY284722
18S	Prodesmodora circulata	AY284719

18S	Prodesmodora circulata	AY284721
18S	Prodesmodora circulata	AY284720
18S	Prodesmodora sp. 1287	FJ040476
18S	Prodesmodora sp. 1338	FJ040477
18S	Prodontorhabditis wirthi	AY602179
18S	Prodorylaimus mas	AY593946
18S	Prodorylaimus sp. HHBM-2007a	EF207246
18S	Prodorylaimus uliginosus	AY284778
18S	Protorhabditis sp. DF5055	AF083001
18S	Protorhabditis sp. JB122	EU196002
18S	Protorhabditis sp. SB208	AF083024
18S	Protostrongylus rufescens	AJ920364
18S	Protozoophaga obesa	EF180075
18S	Pseudacrobeles variabilis	AF202150
18S	Pseudalius inflexus	AY295816
18S	Pseudhalenchus minutus	AY593916
18S	Pseudhalenchus minutus	AY284638
18S	Pseudoterranova decipiens (codworm)	U94380
18S	Psilenchus cf. hilarulus	AY284593
18S	Psilenchus sp. CA12	EU130840
18S	Ptycholaimellus sp. 1092	FJ040472
18S	Punctodera stonei	EU682391
18S	Pungentus silvestris	AY284788
18S	Pungentus sp. PDL-2005	AJ966501
18S	Radopholus similis	AJ966502
18S	Radopholus sp. 1983	FJ040398
18S	Raillietnema sp. V3060	DQ503461
18S	Raphidascaris acus	DQ503460
18S	Rhabditella axei	U13934
18S	Rhabditella axei	AY284654
18S	Rhabditella sp. DF5044	AF083000
18S	Rhabditis blumi	U13935
18S	Rhabditis brassicae	EU196006
18S	Rhabditis cf. terricola JH-2004	AY284653
18S	Rhabditis colombiana	AY751546
18S	Rhabditis myriophila	U13936
18S	Rhabditis myriophila	U81588
18S	Rhabditis rainai	AF083008
18S	Rhabditis sp. DF5059	EU196007
18S	Rhabditis sp. SB347	EU196004
18S	Rhabditis sp. Tumian-2007	EU273597

18S	rhabditoid sp. PDL15	EU196015
18S	Rhabditoid sp. YQ-2006	DQ531722
18S	Rhabditoides regina	AF082997
18S	Rhabditophanes sp. KR3021	AF202151
18S	Rhabdochona denudata	DQ442659
18S	Rhabdolaimus cf. terrestrius JH-2004	AY284710
18S	Rhabdolaimus cf. terrestrius JH-2004	AY284712
18S	Rhabdolaimus cf. terrestrius JH-2004	AY284711
18S	Rhigonema thysanophora	EF180067
18S	Robbea hypermnestra (nomen nudum)	Y16921
18S	Robbea sp. 1 SB-2008	EU768870
18S	Robbea sp. 2 SB-2008	EU768871
18S	Robbea sp. 3 SB-2008	EU784735
18S	Rondonia rondoni	DQ442679
18S	Rotylenchulus reniformis	EU306342
18S	Rotylenchus goodeyi	AY284609
18S	Rotylenchus sp. JH-2004	AY284608
18S	Rotylenchus uniformis	EU306356
18S	Rotylenchus uniformis	AY593882
18S	Ruehmaphelenchus sp. NK202	AB368534
18S	Sabatieria celtica	AY854234
18S	Sabatieria pulchra	EF591335
18S	Sabatieria pulchra	FJ040466
18S	Sabatieria punctata	AY854236
18S	Sabatieria punctata	AY854237
18S	Sabatieria punctata	AY854235
18S	Sabatieria sp. 210-BHMM-2005	AY854238
18S	Sauertylenchus maximus	AY284604
18S	Sauertylenchus maximus	AY284602
18S	Sauertylenchus maximus	AY284603
18S	Schistonchus aureus	DQ912922
18S	Schistonchus centerae	DQ912923
18S	Schistonchus guangzhouensis	DQ912924
18S	Scutellonema bradys	AY271723
18S	Scutellonema bradys	AJ966504
18S	Scutylenchus quadrifer	AY284599
18S	Sectonema barbatoides	AY284814
18S	Sectonema sp. JH-2004	AY284815
18S	Seinura sp. JH-2004	AY284651
18S	Seleborca complexa	U81577
18S	Serratospiculum tendo	AY702704

18S	Setaria digitata	DQ094175
18S	Setaria tundra	EF081341
18S	Setosabatieria hilarula	AY854240
18S	Setostephanolaimus spartinae	EF591321
18S	Skrjabillanus scardinii	DQ442669
18S	Skrjabinema sp. SAN-2007	EF180060
18S	Skrjabingylus chitwoodorum	AY295819
18S	Solididens vulgaris	AY552977
18S	Sphaerolaimus hirsutus	AM234622
18S	Sphaerolaimus hirsutus	AY854228
18S	Sphaerularia bombi	AB250212
18S	Sphaerularia bombi	AB250213
18S	Sphaerularia vespae	AB300595
18S	Spilophorella paradoxa	AY854211
18S	Spinitectus carolini	DQ503464
18S	Spirinia elongata	EF527426
18S	Spirinia elongata	FJ429257
18S	Spirinia parasitifera	AY854217
18S	Spirocamallanus istiblenni	EF180076
18S	Spirocamallanus rarus	DQ494195
18S	Spirocerca lupi	AY751497
18S	Spirocerca sp. SAN-2004	AY751498
18S	Steinernema affine	FJ040425
18S	Steinernema carpocapsae	FJ040416
18S	Steinernema carpocapsae	AF036604
18S	Steinernema carpocapsae	FJ040415
18S	Steinernema feltiae	FJ040418
18S	Steinernema feltiae	FJ040419
18S	Steinernema feltiae	FJ040417
18S	Steinernema glaseri	AY284682
18S	Steinernema glaseri	FJ040422
18S	Steinernema kraussei	FJ040420
18S	Steinernema kraussei	FJ040421
18S	Steinernema monticolum	FJ040423
18S	Steinernema scarabaei	FJ040424
18S	Steinernema sp. 1385	FJ040426
18S	Stenurus minor	AY295817
18S	Stephanurus dentatus	AJ920345
18S	Stilbonema majum	Y16922
18S	Strongyloides callosciureus	AB272229
18S	Strongyloides callosciureus	AB272230

18S	Strongyloides callosciureus	AB453326
18S	Strongyloides cebus	AB272236
18S	Strongyloides fuelleborni	AB453317
18S	Strongyloides fuelleborni fuelleborni	AB272235
18S	Strongyloides mirzai	AB453311
18S	Strongyloides myopotami	AB453313
18S	Strongyloides procyonis	AB205054
18S	Strongyloides procyonis	AB272234
18S	Strongyloides ransomi	AB453327
18S	Strongyloides ratti	AF036605
18S	Strongyloides ratti	U81581
18S	Strongyloides robustus	AB272232
18S	Strongyloides robustus	AB272233
18S	Strongyloides sp. Yufuin-2004	AB453312
18S	Strongyloides stercoralis	AB453315
18S	Strongyloides stercoralis	AF279916
18S	Strongylus equinus	DQ094176
18S	Subanguina radicicola	AF202164
18S	Subanguina radicicola	EU682392
18S	Sulcascaris sulcata	EF180080
18S	Symplocostoma sp. 1279	FJ040502
18S	Syngamus trachea	AJ920344
18S	Syngamus trachea	AF036606
18S	Synhimantus hamatus	EU004819
18S	Synhimantus laticeps	EU004818
18S	Synonchiella sp. 1038	FJ040468
18S	Syphacia muris	EF464553
18S	Syphacia obvelata	EF464554
18S	Syringolaimus sp. 1060	FJ040497
18S	Syringolaimus striatocaudatus	AY854200
18S	Telotylenchus ventralis	AY593905
18S	Teratocephalus lirellus	AF036607
18S	Teratocephalus terrestris	AY284683
18S	Teratorhabditis palmarum	U13937
18S	Teratorhabditis synpapillata	AF083015
18S	Teratorhabditis synpapillata	AB269816
18S	Terranova caballeroi	U94381
18S	Terranova scoliodontis	DQ442661
18S	Terschellingia longicaudata	AM234716
18S	Terschellingia longicaudata	AY854230
18S	Tetrabothriostrongylus mackerrasae	AJ920359

18S	Tetrameres fissipina	EF180077
18S	Thalassoalaimus pirum	FJ040500
18S	Thelastoma krausi	EF180068
18S	Thelazia lacrymalis	DQ503458
18S	Theristus acer	AJ966505
18S	Theristus agilis	AY284694
18S	Theristus agilis	AY284695
18S	Theristus agilis	AY284693
18S	Theristus sp. 1268	FJ040464
18S	Thonus circulifer	AY284795
18S	Thonus minutus	AY284794
18S	Thonus sp. JH-2004	AY284797
18S	Thonus sp. JH-2004	AY284798
18S	Thonus sp. JH-2004	AY284796
18S	Tobrilus gracilis	AJ966506
18S	Torynurus convolutus	AY295818
18S	Toxascaris leonina	U94383
18S	Toxocara canis	AF036608
18S	Toxocara canis	U94382
18S	Toxocara cati	EF180059
18S	Toxocara vitulorum	EF180078
18S	Trichinella britovi	AY851257
18S	Trichinella nativa	AY487254
18S	Trichinella nativa	AY851256
18S	Trichinella nelsoni	AY851261
18S	Trichinella papuae	AY851263
18S	Trichinella pseudospiralis	AY851258
18S	Trichinella sp. T8	AY851262
18S	Trichinella spiralis	U60231
18S	Trichinella spiralis	AY497012
18S	Trichodorus nanjingensis	AJ439579
18S	Trichodorus nanjingensis	AJ439580
18S	Trichodorus pakistanensis	AJ439581
18S	Trichodorus primitivus	AF036609
18S	Trichodorus primitivus	AJ439517
18S	Trichodorus similis	AJ439585
18S	Trichodorus similis	AJ439584
18S	Trichodorus sparsus	AJ439589
18S	Trichodorus variopapillatus	AY284841
18S	Trichostrongylus colubriformis	AJ920350
18S	Trichuris muris	AF036637

18S	Trichuris suis	AY856093
18S	Trichuris suis	AY851265
18S	Trichuris suis	EU790668
18S	Trichuris trichiura	DQ118536
18S	Tridentulus sp. PDL-2005	AJ966507
18S	Tripyla cf. filicaudata JH-2004	AY284731
18S	Tripyla cf. filicaudata JH-2004	AY284730
18S	Tripyla sp. JH-2004	AY284732
18S	Tripyla sp. JH-2004	AY284733
18S	Tripyla sp. JH-2004	AY284734
18S	Tripyla sp. SAN-2007b	EF197730
18S	Tripyla sp. SAN-2007b	EF197731
18S	Tripyla sp. SAN-2007d	EF197735
18S	Tripyla sp. SAN-2007d	EF197734
18S	Tripylella sp. 1031	FJ040488
18S	Tripylina sp. SAN-2007a	EF197727
18S	Tripylina sp. SAN-2007a	EF197729
18S	Tripylina sp. SAN-2007a	EF197728
18S	Tripyloides sp. BHMM-2005	AY854202
18S	Trischistoma monohystera	AJ966509
18S	Trischistoma sp. 1 JH-2004	AY284735
18S	Trischistoma sp. 2 JH-2004	AY284736
18S	Troglostrongylus wilsoni	AY295820
18S	Truttaedacnitis truttae	EF180063
18S	Turbatrix aceti	AF202165
18S	Turgida torresi	EF180069
18S	Tylenchida cf. Helionema sp. MHMH-2008	EU669913
18S	Tylenchidae sp. BHMM-2005	AY854241
18S	Tylenchina sp. WY-433	EU024567
18S	Tylenchina sp. WY-460	EU018049
18S	Tylencholaimellus affinis	AY552978
18S	Tylencholaimellus striatus	AY284837
18S	Tylencholaimus cf. teres HHBM-2007a	EF207254
18S	Tylencholaimus mirabilis	AY284835
18S	Tylencholaimus mirabilis	EF207253
18S	Tylencholaimus sp. JH-2004	AY284834
18S	Tylencholaimus sp. PDL-2005	AJ966510
18S	Tylenchorhynchus claytoni	EU368587
18S	Tylenchorhynchus dubius	EU306352
18S	Tylenchorhynchus dubius	EU368586
18S	Tylenchorhynchus leviterminalis	EU368585

18S	Tylenchorhynchus maximus	AY993979
18S	Tylenchulus semipenetrans	AJ966511
18S	Tylenchus arcuatus	EU306348
18S	Tylenchus arcuatus	EU306349
18S	Tylenchus sp. JH-2003	AY284589
18S	Tylocephalus auriculatus	AF202155
18S	Tylocephalus auriculatus	AY284707
18S	Tylolaimophorus minor	AJ966512
18S	Tylopharynx foetidus	EU306343
18S	uncultured Diplolaimelloides	EF659927
18S	uncultured Diplolaimelloides	EF659919
18S	uncultured Diplolaimelloides	EF659918
18S	uncultured Diplolaimelloides	EF659925
18S	uncultured Diplolaimelloides	EF659926
18S	uncultured Diplolaimelloides	EF659917
18S	uncultured Diplolaimelloides	EF659924
18S	Viscosia sp. 1267	FJ040494
18S	Viscosia sp. BHMM-2005	AY854197
18S	Viscosia viscosa	AY854198
18S	Wellcomia siamensis	EF180079
18S	Wellcomia sp. SAN-2007	EF180066
18S	Wilsonema otophorum	AY593927
18S	Wilsonema schuurmansstekhoveni	AJ966513
18S	Wuchereria bancrofti	AF227234
18S	Wuchereria bancrofti	AY843438
18S	Xiphidorus balcarceanus	AY297839
18S	Xiphidorus minor	AY297830
18S	Xiphidorus minor	AY604181
18S	Xiphidorus parthenus	AY604182
18S	Xiphidorus sp. 1 CMGO-2004	AY604183
18S	Xiphidorus sp. RN-2003	AY297841
18S	Xiphidorus yepesara parthenus	AY297837
18S	Xiphidorus yepesara yepesara	AY297838
18S	Xiphinema americanum	AY283170
18S	Xiphinema americanum	AY580056
18S	Xiphinema brasiliense	AY297836
18S	Xiphinema cf. americanum RN-2005	AM086671
18S	Xiphinema cf. americanum RN-2005	AM086679
18S	Xiphinema cf. americanum RN-2005	AM086683
18S	Xiphinema diffusum	AM086677
18S	Xiphinema diversicaudatum	EF538761

18S	Xiphinema elongatum	AY297824
18S	Xiphinema georgianum	AM086688
18S	Xiphinema incognitum	AM086678
18S	Xiphinema incognitum	AM086670
18S	Xiphinema index	EF207249
18S	Xiphinema longicaudatum	AY297829
18S	Xiphinema paritaliae	AY297831
18S	Xiphinema rivesi	AF036610
18S	Xiphinema surinamense	AY297833
18S	Xyzzors sp.	Y16923
18S	Zeldia punctata	U61760
18S	Zeldia sp. JH-2004	AY284676
18S	Zeldia sp. JH-2004	AY284675
18S	Zoniolaimus mawsonae	AJ920338
18S	Zygotylenchus guevarae	AF442189
28S	Acrobeles complexus	DQ145620
28S	Acrobeles maeneeneus	DQ145621
28S	Acrobeles maximus	EU195987
28S	Acrobeles singulus	DQ145622
28S	Acrobeles sp. JB-132	DQ145623
28S	Acromoldavicus aff. mojavicus SAN-2001	AY027534
28S	Acromoldavicus mojavicus	DQ145626
28S	Acrobeloides bodenheimeri	AF147065
28S	Acrobeloides bodenheimeri	AF147064
28S	Acrobeloides bodenheimeri	DQ145625
28S	Acrobeloides buetschlii	DQ903104
28S	Acrobeloides buetschlii	DQ903081
28S	Acrobeloides camberenensis	DQ903088
28S	Acrobeloides camberenensis	AF147069
28S	Acrobeloides ellesmerensis	DQ145624
28S	Acrobeloides maximus	AF147066
28S	Acrobeloides maximus	AF147067
28S	Acrobeloides maximus	DQ903097
28S	Acrobeloides maximus	EF417138
28S	Acrobeloides maximus	DQ903078
28S	Acrobeloides nanus	EF417139
28S	Acrobeloides nanus	DQ903075
28S	Acrobeloides nanus	DQ903076
28S	Acrobeloides nanus	DQ903103
28S	Acrobeloides sp. PP2	DQ077788
28S	Acrobeloides sp. JB-68	DQ903091

28S	Acrobeloides sp. JB-80	DQ903096
28S	Acrobeloides sp. DWF-1106	DQ903080
28S	Acrobeloides sp. JB-77	DQ903093
28S	Acrobeloides sp. PS-1146	DQ903101
28S	Acrobeloides sp. DWF-1108	DQ903082
28S	Acrobeloides sp. IZ-001	DQ903085
28S	Acrobeloides sp. JB-14	DQ903086
28S	Acrobeloides sp. PDL-33	DQ903099
28S	Acrobeloides sp. DWF-1105	DQ903079
28S	Acrobeloides thornei	AF147068
28S	Acrobeloides thornei	DQ903083
28S	Acrobeloides uberrinus	DQ903087
28S	Acrostichus halicti	EU195983
28S	Aelurostrongylus abstrusus	AM039759
28S	Alaimus sp. PDL-2005	DQ077791
28S	Allodorylaimus andrassyi	AY593016
28S	Allodorylaimus andrassyi	AY593015
28S	Amidostomum cygni	AM039745
28S	Amplimerlinius icarus	DQ328714
28S	Anatonchus tridentatus	AY593065
28S	Ancylostoma caninum (dog hookworm)	AM039739
28S	Anguina tritici	DQ328723
28S	Anisakis simplex 'C' SAN-2004	AY821754
28S	Anisakis sp. SAN-2004	AY821759
28S	Angiostrongylus cantonensis	AY292792
28S	Angiostrongylus vasorum	AM039758
28S	Angiostrongylus vasorum	AM039758
28S	Aphelenchoides besseyi	DQ328684
28S	Aphelenchoides besseyi	AY508109
28S	Aphelenchoides fragaria	DQ328683
28S	Aphelenchoides fragariae	AB368540
28S	Aphelenchoides sp. TG102006	EU084037
28S	Aphelenchoides sp. CA22	DQ328682
28S	Aphelenchoides stammeri	AM396582
28S	Aphelenchus avenae	AB368536
28S	Aphelenchus sp. SAN-2005	DQ145664
28S	Aporcelaimellus obtusicaudatus	AY593018
28S	Aporcelaimellus obtusicaudatus	AY593019
28S	Aporcelaimellus cf. paraobtusicaudatus JH-2004	AY593020
28S	Aporcelaimellus cf obtusicaudatus JH-2004	AY593017
28S	Aporcelaimellus sp. JH-2004	AY593021

28S	Ascaris suum (pig roundworm)	AY821773
28S	Ascaris lumbricoides (common roundworm)	AY210806
28S	Ascolaimus sp. 1P6K2	DQ077749
28S	Atalodera crassicrustata	DQ328704
28S	Axonchium propinquum	AY593022
28S	Basiria gracilis	DQ328717
28S	Basiria sp. SAN-2005	DQ145619
28S	Baylisascaris procyonis	AY821774
28S	Belonolaimus euthychilus	DQ672359
28S	Belonolaimus euthychilus	DQ672360
28S	Belonolaimus euthychilus	DQ672361
28S	Belonolaimus gracilis	DQ672362
28S	Belonolaimus gracilis	DQ672363
28S	Belonolaimus longicaudatus	DQ672354
28S	Belonolaimus longicaudatus	DQ672345
28S	Belonolaimus longicaudatus	DQ672356
28S	Belonolaimus longicaudatus	DQ672347
28S	Belonolaimus longicaudatus	DQ672358
28S	Belonolaimus longicaudatus	DQ672343
28S	Belonolaimus longicaudatus	DQ672355
28S	Belonolaimus longicaudatus	DQ672349
28S	Belonolaimus longicaudatus	DQ672353
28S	Belonolaimus longicaudatus	DQ672352
28S	Belonolaimus longicaudatus	DQ672346
28S	Belonolaimus longicaudatus	DQ672348
28S	Belonolaimus longicaudatus	DQ672350
28S	Belonolaimus longicaudatus	DQ915803
28S	Belonolaimus longicaudatus	DQ672344
28S	Belonolaimus longicaudatus	DQ672357
28S	Belonolaimus longicaudatus	DQ672351
28S	Boleodorus sp. Germany 709	DQ328718
28S	Bradynema listronotum	DQ915804
28S	Bradynema rigidum	DQ328730
28S	Brevibucca punctata	DQ077787
28S	Brevibucca saprophaga	DQ077786
28S	Brevibucca saprophaga	EU195990
28S	Brevibucca sp. SB-261	DQ145627
28S	Bursilla sp. PS1179	EF990722
28S	Bursilla sp. PS1179	EF990722
28S	Bunonema reticulatum	EU195989
28S	Bunonema sp. PDL-2005	DQ077789

28S	Bursaphelenchus abietinus	AY508074
28S	Bursaphelenchus arthuri	AM396564
28S	Bursaphelenchus hylobianum	AY508085
28S	Bursaphelenchus mucronatus	EU295500
28S	Bursaphelenchus mucronatus	EU295493
28S	Bursaphelenchus parvispicularis	AB368537
28S	Bursaphelenchus rainulfi	AM396575
28S	Bursaphelenchus doui	AB299226
28S	Bursaphelenchus sexdentati	AY508100
28S	Bursaphelenchus sexdentati	AY508103
28S	Bursaphelenchus sinensis	EU752257
28S	Bursaphelenchus sp. 169	AY508092
28S	Bursaphelenchus sp. P4193	EU159108
28S	Bursaphelenchus tusciae	AY508104
28S	Bursaphelenchus xylophilus	EU295491
28S	Cactodera cacti	DQ328702
28S	Caenorhabditis brenneri	DQ059062
28S	Caenorhabditis brenneri	AY602175
28S	Caenorhabditis briggsae	EF417140
28S	Caenorhabditis briggsae	AY604481
28S	Caenorhabditis briggsae	AY604481
28S	Caenorhabditis drosophilae	AY602172
28S	Caenorhabditis elegans	EF417141
28S	Caenorhabditis elegans	X03680
28S	Caenorhabditis japonica	AY602173
28S	Caenorhabditis plicata	AY602167
28S	Caenorhabditis remanei	AY602174
28S	Caenorhabditis sp. SB341	AY602170
28S	Caenorhabditis sp. JU727	EU195957
28S	Caenorhabditis sp. SB341	AY602170
28S	Caenorhabditis sp. DF5070	AY602171
28S	Caenorhabditis sp. JU727	EU195957
28S	Caenorhabditis sp. PS1010	AY604482
28S	Caenorhabditis sp. DF5070	AY602171
28S	Carcharodiscus banaticus	AY593023
28S	Carcharodiscus banaticus	AY593024
28S	Cryphodera brinkmani	DQ328705
28S	Cephalobidae sp. PS1146	EF417142
28S	Cervidellus alutus	DQ145629
28S	Cervidellus alutus	AF331911
28S	Cervidellus doorsselaeri	DQ145630

28S	Cervidellus neftasiensis	DQ145631
28S	Cervidellus sp. JB-138	DQ145632
28S	Chabertia ovina	AM039733
28S	Choriorhabditis cristata	EU195976
28S	Choriorhabditis dudichi	EU195975
28S	Chiloplacus sp. JB-81	DQ145634
28S	Chromadorina sp. 1M21G4	DQ077776
28S	Choanolaimus sp. 8M21G4	DQ077777
28S	Chrysonema attenuatum	AY593029
28S	Clavicaudoides clavicaudatus	EF207234
28S	Clavicaudoides clavicaudatus	EF207235
28S	Clavicaudoides trophurus	EF207237
28S	Clavicaudoides trophurus	EF207236
28S	Cylicocyclus insignis	AM039734
28S	Chromadorida sp. 9P8K2	DQ077765
28S	Chromadorida sp. 1I11K2	DQ077757
28S	Chromadorida sp. 5I9K2	DQ077755
28S	Ceramonematidae sp. 8I12K2	DQ077773
28S	Cranifera cranifera	EU365632
28S	Contracaecum eudyptulae	AF226586
28S	Contracaecum micropapillatum	AF226587
28S	Contracaecum microcephalum	AF226573
28S	Contracaecum mirounga	AF226581
28S	Contracaecum multipapillatum	AF226574
28S	Contracaecum ogmorhini	AF226582
28S	Contracaecum osculatum baicalensis	AF226589
28S	Contracaecum osculatum	AF226583
28S	Contracaecum osculatum	AF226576
28S	Contracaecum osculatum	AF226580
28S	Contracaecum radiatum	AF226577
28S	Contracaecum rudolphii	AF226579
28S	Contracaecum rudolphii	AF226585
28S	Contracaecum septentrionale	AF226588
28S	Contracaecum sp. SAN-2004	AY821768
28S	Contracaecum sp. SAN-2004	AY821770
28S	Contracaecum sp. SAN-2004	AY821769
28S	Coslenchus costatus	DQ328719
28S	Cephalobus cubaensis	DQ903102
28S	Cephalobus persegnis	DQ903077
28S	Cephalobus sp. JB-65	DQ903090
28S	Cephalobus sp. JB-63	DQ903089

28S	Cephalobus sp. DWF-1301	DQ903084
28S	Cephalobus sp. JB-78	DQ903094
28S	Cephalobus sp. JB-117	DQ903098
28S	Cephalobus sp. JB-67	DQ145628
28S	Cephalobus sp. JB-70	DQ903092
28S	Cephalobus sp. JB-79	DQ903095
28S	Cephalobus sp. PS-1143	DQ903100
28S	Cephaloboides cf. armata SB363	EU195961
28S	Cephaloboides nidrosiensis	EU195992
28S	Crenosoma mephitidis	AY292793
28S	Crenosoma vulpis	AM039760
28S	Crenosoma vulpis	AM039760
28S	Crustorhabditis transita	EU195995
28S	Cruznema tripartitum	EU195974
28S	Cystoopsis atractostei	DQ060331
28S	Cuticonema vivipara	EU195991
28S	Cyatholaimidae sp. 2I12K3	DQ077782
28S	Cyclodontostomum purvisi	AM039732
28S	Dictyocaulus filaria	AM039754
28S	Dictyocaulus filaria	AM039754
28S	Dictyocaulus viviparus (bovine lungworm)	AM039753
28S	Deladenus siricidicola	AY633444
28S	Dicelis lovatiana	AY967868
28S	Dicelis rubidi	AY967866
28S	Dicelis sp. 'Aberdeen'	AY967867
28S	Didelphostrongylus hayesi	AY292794
28S	Discolaimus cf. major HHBM-2007a	EF207239
28S	Discolaimus major	AY593025
28S	Discolaimus major	AY593026
28S	Dolichodera sp. New Zealand 576	DQ328701
28S	Deletrocephalus dimidiatus	AM039738
28S	Dolichodorus mediterraneus	DQ838803
28S	Diploscapter sp. JU359	EU195959
28S	Diplogaster ineritieri	EF417143
28S	Diphtherophora sp. PDL-2005	DQ077790
28S	Dorylaimoides limnophilus	AY593003
28S	Dorylaimoides micoletzkyi	AY593004
28S	Dorylaimus stagnalis	AY592995
28S	Dorylaimus stagnalis	AY592994
28S	Discolaimoides symmetricus	EF207238
28S	Distolabrellus veechi	EF990725

28S	Eucephalobus sp. JB-55	DQ145635
28S	Ecumenicus monohystera	AY593013
28S	Ecumenicus sp. JH-2004	AY593014
28S	Ektaphelenchoides compasi	DQ257625
28S	Ektaphelenchoides pini	DQ257623
28S	Ektaphelenchus obtusus	AB368533
28S	Enchodelus macrodorus	AY593054
28S	Enchodelus sp. HHBM-2007a	EF207240
28S	Enoplolaimus sp. 2P6K2	DQ077750
28S	Enoploides sp. 3I11K2	DQ077759
28S	Enoploides sp. 1P11K2	DQ077760
28S	Enoploides sp. 2P9K2	DQ077764
28S	Epidorylaimus lugdunensis	AY593036
28S	Epidorylaimus lugdunensis	AY593035
28S	Eudorylaimus centrocercus	AY593007
28S	Eudorylaimus sp. JH-2004	AY593037
28S	Eumonhystera filiformis	DQ086658
28S	Fescia grossa	DQ145636
28S	Filaroides martis	AY292795
28S	Filarinema flagrifer	AM039746
28S	Globodera artemisiae	EU855121
28S	Globodera millefolii	DQ328700
28S	Globodera pallida	AY592991
28S	Globodera pallida	AY592992
28S	Globodera pallida	EU855119
28S	Globodera rostochiensis	AY592988
28S	Globodera rostochiensis	AY592987
28S	Globodera rostochiensis	AY592993
28S	Globodera rostochiensis	EU855120
28S	Haemonchus contortus	AM039742
28S	Haemonchus sp. 92619	AY292796
28S	Haliplectus sp. 7I16G4	DQ077774
28S	Hammerschmidtiella cristata	EU365629
28S	Helicotylenchus multicinctus	DQ328746
28S	Helicotylenchus multicinctus	DQ328745
28S	Helicotylenchus pseudorobustus	DQ328750
28S	Helicotylenchus pseudorobustus	DQ328751
28S	Helicotylenchus pseudorobustus	DQ328747
28S	Helicotylenchus pseudorobustus	DQ328748
28S	Helicotylenchus pseudorobustus	DQ328749
28S	Hemicycliophora sp. Vovlas-IPP	AY780974

28S	Herpetostrongylus pythonis	AM039750
28S	Heterodera aucklandica	DQ328688
28S	Heterodera cajani	DQ328693
28S	Heterodera cynodontis	DQ328698
28S	Heterodera zeae	DQ328695
28S	Heterodera glycines	DQ328692
28S	Heterodera goettingiana	DQ328697
28S	Heterodera koreana	EU284032
28S	Heterodera latipons	DQ328687
28S	Heterodera litoralis	DQ328691
28S	Heterodera orientalis	EU284033
28S	Heterodera oryzicola	DQ328694
28S	Heterodera salixophila	DQ328690
28S	Heterodera sorghi	DQ328689
28S	Heterodera sp. SAS-2008	EU284031
28S	Heterodera urticae	DQ328696
28S	Hirschmanniella pomponiensis	DQ077795
28S	Hirschmanniella santarosae	EF029859
28S	Hirschmanniella cf. belli ITDL-2006	EF029860
28S	Hirschmanniella sp. VietNam Chau	DQ328686
28S	Hirschmanniella sp. Yuma	EF029861
28S	Halicephalobus gingivalis	AB289346
28S	Halicephalobus gingivalis	AY294178
28S	Halicephalobus gingivalis	AY294181
28S	Halicephalobus gingivalis	AY294177
28S	Halicephalobus gingivalis	AB288935
28S	Halicephalobus gingivalis	AY294182
28S	Halicephalobus gingivalis	AY294179
28S	Halicephalobus gingivalis	AB289345
28S	Halicephalobus gingivalis	AY294180
28S	Heligmosomoides polygyrus	AM039747
28S	Hoplolaimus columbus	EU554668
28S	Hoplolaimus columbus	EU554674
28S	Hoplolaimus columbus	EU554669
28S	Hoplolaimus columbus	EU554670
28S	Hoplolaimus concaudajuvencus	EU626792
28S	Hoplolaimus galeatus	EU626785
28S	Hoplolaimus galeatus	EU626784
28S	Hoplolaimus magnistylus	EU626789
28S	Hoplolaimus seinhorsti	EU626791
28S	Hoplolaimus sp. 1 CB-2008	EU626793

28S	Hoplolaimus sp. 2 CB-2008	EU626795
28S	Hoplolaimus sp. 3 CB-2008	EU586797
28S	Howardula phyllotretae	DQ328728
28S	Heterorhabditidoides chongmingensis	EF503691
28S	Heterocheilus tunicatus	AF226592
28S	Heterorhabditis amazonensis	EU099036
28S	Heterorhabditis bacteriophora	EU313541
28S	Heterorhabditis bacteriophora	EU099037
28S	Heterorhabditis floridensis	EU099034
28S	Heterorhabditis georgiana	EU099033
28S	Heterorhabditis indica	EU100415
28S	Heterorhabditis marelatus	DQ145665
28S	Heterorhabditis marelatus	EU100412
28S	Heterorhabditis megidis	EU100413
28S	Heterorhabditis mexicana	EU100414
28S	Heterorhabditis safricana	EU100416
28S	Heterorhabditis sp. DHAF-2007a	EU195993
28S	Heterorhabditis zealandica	EU099035
28S	Heterorhabditis zealandica	AM039761
28S	Heterorhabditis zealandica	AM039761
28S	Heteroanguina graminophila	DQ328720
28S	Heterocephalobellus sp. JB-8	DQ145638
28S	Hypodontus macropi	AM039731
28S	Hysterothylacium auctum	AF226591
28S	Hysterothylacium pelagicum	AF226590
28S	Kalicephalus cristatus	AM039741
28S	Koerneria sp. 228	AY840563
28S	Koerneria sp. SB110	EU195999
28S	Latronema sp. 2P15K2	DQ077784
28S	Latronema sp. 1P10K3	DQ077780
28S	Labronema vulvapapillatum	AY592996
28S	Labronema vulvapapillatum	AY592997
28S	Labiostrongylus bipapillosus	AJ512837
28S	Leidynema appendiculata	EU365630
28S	Laimaphelenchus sp. RGD636L	AB368539
28S	Longidorus americanum	AY494715
28S	Longidorus caespiticola	AF480080
28S	Longidorus caespiticola	AF480079
28S	Longidorus caespiticola	AF480081
28S	Longidorus carpathicus	AF480072
28S	Longidorus elongatus	AF480077

28S	Longidorus elongatus	AF480075
28S	Longidorus elongatus	AF480076
28S	Longidorella cf macramphis JH-2004	AY593042
28S	Longidorus intermedius	AF480074
28S	Longidorus macrosoma	AF480082
28S	Longidorus profundorum	AF480073
28S	Longidorella sp. 1 JH-2004	AY593045
28S	Longidorella sp. 3 JH-2004	AY593044
28S	Longidorella sp. 2 JH-2004	AY593043
28S	Longidorus sturhanii	AF480071
28S	Longidorus uroshis	EF538754
28S	Macrotrophurus arbusticola	DQ328708
28S	Macrolaimellus sp. SAN-2005	DQ145640
28S	Macrolaimus sp. SAN-2005	DQ145639
28S	Meloidogyne arenaria	U42339
28S	Meloidogyne arenaria	AF435803
28S	Meloidogyne arenaria	EU364889
28S	Meloidogyne artiellia	AY150369
28S	Meloidogyne baetica	AY150367
28S	Meloidogyne chitwoodi	AF435802
28S	Meloidogyne dunensis	EF612712
28S	Meloidogyne exigua	AF435804
28S	Meloidogyne exigua	AF435796
28S	Meloidogyne exigua	AF435795
28S	Meloidogyne graminicola	AF435793
28S	Meloidogyne hapla	DQ328685
28S	Meloidogyne hispanica	EU443608
28S	Meloidogyne hispanica	EU443607
28S	Meloidogyne hispanica	EU443606
28S	Meloidogyne ichinohei	EF029862
28S	Meloidogyne incognita	AF435794
28S	Meloidogyne konaensis	AF435797
28S	Meloidogyne paranaensis	AF435800
28S	Meloidogyne paranaensis	AF435799
28S	Meloidogyne paranaensis	AF435798
28S	Meloidogyne thailandica	EU364890
28S	Meloidogyne trifoliophila	AF435801
28S	Metachromadora sp. 2I14K2	DQ077783
28S	Metachromadora sp. 4P6K2	DQ077752
28S	Microdorylaimus miser	AY593046
28S	Microdorylaimus modestus	AY593049

28S	Meloidoderita kirjanovae	DQ768428
28S	Meloidodera alni	DQ328706
28S	Monhysterida sp. 3P12K2	DQ077767
28S	Mononchus truncatus	AY593064
28S	Mononchus tunbridgensis	AY593063
28S	Mesodorylaimus sp. JH-2004	AY593006
28S	Mesodorylaimus sp. JH-2004	AY593005
28S	Mesoanguina millefolii	DQ328722
28S	Mesorhabditis anisomorpha	EF990723
28S	Mesorhabditis longespiculosa	EU195980
28S	Metacrobeles amblyurus	DQ145642
28S	Metastrongylus elongatus	AM039755
28S	Metastrongylus salmi	AY292797
28S	Muellerius capillaris	AY292798
28S	Myctolaimus ulmi	EU195998
28S	Myctolaimus ulmi	EU195998
28S	Myolaimus sp. RGD233	DQ145643
28S	Nagelus leptus	DQ328715
28S	Necator americanus	AF217868
28S	Necator americanus	AM039740
28S	Nematodirus battus	AY292799
28S	Nematodirus battus	AM039752
28S	Nematodirus battus	AM039752
28S	Neodiplogaster crenatae	AB326309
28S	Nicollina cameroni	AM039749
28S	Nippostrongylus brasiliensis	AM039748
28S	Nothacrobeles borregi	DQ145645
28S	Nothacrobeles spatulatus	DQ145644
28S	Nothacrobeles spatulatus	AY027532
28S	Nothacrobeles triniglarus	DQ145646
28S	Neotylenchus sp. SAS-2006	DQ328725
28S	Nygolaimus cf. brachyuris JH-2004	AY593061
28S	Odontophora sp. 5P9K2	DQ077756
28S	Odontophora sp. 2I11K2	DQ077758
28S	Odontopharynx longicaudata	DQ077775
28S	Oncholaimidae sp. 2I6K2	DQ077753
28S	Opisthodorylaimus sylphoides	AY593008
28S	Opisthodorylaimus sylphoides	AY593009
28S	Opisthodorylaimus sylphoides	AY593010
28S	Oscheius dolichuroides	EU195970
28S	Oscheius dolichura	EU195971

28S	Oscheius guentheri	EU195996
28S	Oscheius insectivora	EU195968
28S	Oscheius myriophila	AY602176
28S	Oscheius tipulae	DQ059063
28S	Oscheius tipulae	EU195969
28S	Oslerus osleri	AY292800
28S	Ostertagia leptospicularis	AM039744
28S	Otostrongylus circumlitus	AY292801
28S	Oxydirus nethus	AY593011
28S	Oxydirus oxycephalus	AY593012
28S	Panagrolaimus sp. 2 PS1159	DQ059061
28S	Panagrolaimus sp. RS-2007a	EF417144
28S	Panagrolaimus sp. R18	EF417146
28S	Panagrolaimus sp. RS-2007b	EF417145
28S	Panagrolaimus sp. JB115	AY294183
28S	Panagrolaimus sp. SAN-15	DQ145651
28S	Paravulvus hartingii	AY593062
28S	Passalurus ambiguus	EF464552
28S	Paractinolaimus macrolaimus	AY593000
28S	Paractinolaimus macrolaimus	AY592999
28S	Paractinolaimus macrolaimus	AY592998
28S	Prodontorhabditis wirthi	AY602169
28S	Prodorylaimus sp. HHBM-2007a	EF207241
28S	Prodorylaimus uliginosus	AY593034
28S	Pelodera cylindrica	EU195994
28S	Pellioditis marina	AM937040
28S	Pellioditis marina	AM399044
28S	Pellioditis marina	AM399038
28S	Pellioditis marina	AM399055
28S	Pellioditis marina	AM399063
28S	Pellioditis marina	AM937034
28S	Pellioditis marina	AM399043
28S	Pellioditis marina	AM937038
28S	Pellioditis marina	AM399062
28S	Pellioditis marina	AM399039
28S	Pellioditis marina	AM399050
28S	Pelodera pseudoteres	EU195997
28S	Pelodera punctata	EU195978
28S	Pelodera strongyloides	EU195977
28S	Pelodera teres	EU195979
28S	Petrovinema poculatum	AM039735

28S	Phasmarhabditis sp. EM434	EU195967
28S	Phanoderma sp. 3I23B4	DQ077781
28S	Phanoderma sp. 5I23B4	DQ077769
28S	Phocascaris cystophorae	AF226578
28S	Phocascaris phocae	AF226584
28S	Phocascaris sp. 112000	AF226575
28S	Plectonchus hunti	DQ145652
28S	Plectidae sp. SAS-2004	AY652779
28S	Plectus aquatilis	EF417147
28S	Plectus minimus	EF417148
28S	Paralongidorus maximus	AF480083
28S	Paralongidorus paramaximus	EU026156
28S	Parelaphostrongylus odocoilei	AY292803
28S	Peltamigratus perscitus	DQ328744
28S	Punctodera punctata	DQ328699
28S	Panagrellus ceylonensis	DQ408251
28S	Panagrellus dubius	DQ408258
28S	Panagrellus dubius	DQ408253
28S	Panagrellus dubius	DQ408257
28S	Panagrellus dubius	DQ408256
28S	Panagrellus dubius	DQ408254
28S	Panagrellus dubius	DQ408255
28S	Panagrellus dubius	DQ408252
28S	Panagrellus redivivus	DQ145647
28S	Panagrellus redivivus	DQ408249
28S	Panagrellus redivivus	AF331910
28S	Panagrellus redivivus	DQ408250
28S	Panagrellus redivivus	EU195986
28S	Panagrobelus stammeri	DQ145649
28S	Poikilolaimus ernstmayri	DQ059058
28S	Poikilolaimus oxycercus	DQ059059
28S	Poikilolaimus oxycercus	EU195984
28S	Poikilolaimus regenfussi	DQ059057
28S	Poikilolaimus sp. RGD617	AB370213
28S	Pomponema sp. 2P12K2	DQ077763
28S	Pontonema sp. 6I23B4	DQ077771
28S	Pontonema sp. 3I24B4	DQ077768
28S	Protorhabditis sp. DF5055	AY602168
28S	Protorhabditis sp. JB122	EU195958
28S	Paracanthonchus sp. 4I6K2	DQ077754
28S	Parafilaroides decorus	AY292802

28S	Parafilaroides decorus	AM039757
28S	Prionchulus sp. DGW_GPhi	DQ077802
28S	Pratylenchus brzeskii	AM231927
28S	Pratylenchus brzeskii	AM231912
28S	Pratylenchus brzeskii	AM231920
28S	Pratylenchus coffeae	EU130850
28S	Pratylenchus coffeae	AF170429
28S	Pratylenchus dunensis	AM231946
28S	Pratylenchus dunensis	AM231939
28S	Pratylenchus dunensis	AM231948
28S	Pratylenchus gutierrezi	AF170442
28S	Pratylenchus loosi	EF446994
28S	Pratylenchus loosi	EF446992
28S	Pratylenchus zeae	EU130896
28S	Pratylenchus zeae	EU130894
28S	Pratylenchus penetrans	EU130859
28S	Pratylenchus penetrans	EU130860
28S	Pratylenchus pratensis	AM231934
28S	Pratylenchus thornei	EU130880
28S	Pratylenchus vulnus	EU130887
28S	Pratylenchus vulnus	EU130882
28S	Pristionchus lheritieri	DQ059066
28S	Pristionchus maupasi	DQ059065
28S	Pristionchus pacificus	DQ059064
28S	Pristionchus pacificus	EU195982
28S	Pristionchus sp. RS141	AF549407
28S	Paratrichodorus anemones	AJ781505
28S	Paratrichodorus pachydermus	AM180727
28S	Paratrichodorus porosus	EU827614
28S	Paratrichodorus renifer	EU827615
28S	Paraxonchium laetificans	AY593001
28S	Pseudacrobeles sp. JB-85	DQ145654
28S	Pseudacrobeles sp. JB-56	DQ145653
28S	Pseudacrobeles variabilis	AF143368
28S	Pseudalius inflexus	AY292804
28S	Pseudoterranova decipiens (codworm)	AY821761
28S	Pseudoterranova decipiens (codworm)	AY821760
28S	Pseudoterranova decipiens (codworm)	AY821762
28S	Pseudoterranova decipiens (codworm)	AY821763
28S	Psilenchus sp. USA CA9	DQ328716
28S	Parasitylenchus sp. SAS-2006	DQ328729

28S	Parasitodiplogaster citrinema	AY840555
28S	Parasitodiplogaster laevigata	AY840558
28S	Parasitodiplogaster laevigata	AY840557
28S	Parasitodiplogaster laevigata	AY840556
28S	Parasitodiplogaster maxinema	AY840559
28S	Parasitodiplogaster popenema	AY840560
28S	Parasitodiplogaster sp. WY-579p	EU018054
28S	Parasitodiplogaster sp. WY-463p	EU018051
28S	Parasitodiplogaster sp. 239	AY840561
28S	Parasitodiplogaster trigonema	AY840562
28S	Parascaris equorum	AY821775
28S	Parasitorhabditis obtusa	EF990724
28S	Pratylenchidae sp. Trinh 104108	EF645137
28S	Pratylenchidae sp. Trinh 104107	EF645138
28S	Protostrongylus rufescens	AM039756
28S	Pungentus engadinensis	AY593050
28S	Pungentus silvestris	AY593053
28S	Pungentus silvestris	AY593052
28S	Radopholus sp. 7B VietNam	DQ328712
28S	Raphidascaris acus	AY821772
28S	Richtersia sp. 4P11K2	DQ077762
28S	Richtersia sp. 5P12K2	DQ077770
28S	Rhabditoides inermis	EU195981
28S	Rhabditoides inermiformis	EF990727
28S	Rhabditoides regina	EF990726
28S	Rhabditis brassicae	EU195963
28S	Rhabditis dolichura	EF417150
28S	Rhabditis rainai	EU195966
28S	Rhabditis sp. BC7735	EU303298
28S	Rhabditis sp. SB347	EU195960
28S	Rhabditis sp. DF5059	EU195964
28S	Rhabdocoma sp. 1I12K3	DQ077778
28S	Rhabdias bakeri	EU360836
28S	Rhabdias bakeri	EU360833
28S	Rhabdias bakeri	DQ264774
28S	Rhabdias bakeri	DQ264773
28S	Rhabdias bakeri	EU360835
28S	Rhabdias pseudosphaerocephala	DQ845736
28S	Rhabdias pseudosphaerocephala	DQ845737
28S	Rhabdias pseudosphaerocephala	DQ845735
28S	Rhabdias ranae	DQ264768

28S	Rhabdias ranae	DQ264769
28S	Rhabdias ranae	EU360844
28S	Rhabdias ranae	EU360842
28S	Rhabdias ranae	EU360843
28S	Rhabdias cf. hylae SD-2008	EU836866
28S	Rhabdias cf. hylae SD-2008	EU836874
28S	Rhabdias sp. SD-2008	EU836870
28S	Rhabdias sphaerocephala	DQ845739
28S	Rhabdias sphaerocephala	DQ845741
28S	Rhabditella axei	AY602177
28S	Rhabditophanes sp. KR3021	AY294185
28S	Rhabditophanes sp. KR3021	DQ145655
28S	rhabditoid sp. PDL15	EU195985
28S	Rhizonema sequoiae	DQ328703
28S	Romanomermis culicivorax	EF417153
28S	Rotylenchulus macrodoratus	DQ328711
28S	Rotylenchulus reniformis	DQ328713
28S	Rotylenchus cazorlaensis	EU280792
28S	Rotylenchus cazorlaensis	EU280793
28S	Rotylenchus eximius	EU280794
28S	Rotylenchus eximius	DQ328741
28S	Rotylenchus goodeyi	DQ328756
28S	Rotylenchus incultus	EU280797
28S	Rotylenchus incultus	EU280796
28S	Rotylenchus jaeni	EU280791
28S	Rotylenchus laurentinus	EU280798
28S	Rotylenchus laurentinus	DQ328757
28S	Rotylenchus magnus	EU280790
28S	Rotylenchus magnus	EU280789
28S	Rotylenchus robustus	EU280788
28S	Rotylenchus uniformis	DQ328737
28S	Rotylenchus uniformis	DQ328738
28S	Rotylenchus uniformis	DQ328735
28S	Rotylenchus uniformis	DQ328740
28S	Rotylenchus uniformis	DQ328736
28S	Rotylenchus uniformis	DQ328739
28S	Rotylenchus unisexus	EU280799
28S	Ruehmaphelenchus asiaticus	AM269475
28S	Schistonchus aureus	DQ912925
28S	Schistonchus centerae	DQ912928
28S	Schistonchus guangzhouensis	DQ912927

28S	Schistonchus laevigatus	DQ912926
28S	Schistonchus sp. WY-463s	EU018052
28S	Sectonema barbatoides	AY593032
28S	Sectonema barbatoides	AY593031
28S	Sectonema barbatoides	AY593030
28S	Sectonema sp. JH-2004	AY593033
28S	Severianoia sp. 1 SS-2008	EU365631
28S	Skrjabingylus chitwoodorum	AY292805
28S	Syngamus trachea	AM039736
28S	Sphaerularia bombi	DQ328726
28S	Steinernema abbasi	AF331890
28S	Steinernema arenarium	AF331892
28S	Steinernema ceratophorum	AF331888
28S	Steinernema cubanum	AF331889
28S	Steinernema intermedium	AF331909
28S	Steinernema khoisanae	DQ314289
28S	Steinernema kraussei	AF331896
28S	Steinernema kushidai	AF331897
28S	Steinernema longicaudum	AF331901
28S	Steinernema rarum	DQ221118
28S	Steinernema scarabaei	AY172023
28S	Steinernema sp. SS-2007a	EU177771
28S	Steinernema sp. 1 'Costa Rica'	EF187017
28S	Steinernema weiseri	DQ400854
28S	Steinernema yirgalemense	AY748451
28S	Stegelletina similis	AY027533
28S	Stegelletina sp. JB-139	DQ145659
28S	Stegelletina sp. JB-64	DQ145658
28S	Stegelletina sp. SAN-2005	DQ145657
28S	Stephanurus dentatus	AM039737
28S	Stephanurus dentatus	AM039737
28S	Strongyloides callosciureus	AB272229
28S	Strongyloides callosciureus	AB272230
28S	Strongyloides callosciureus	AB272231
28S	Strongyloides fuelleborni	U42595
28S	Strongyloides fuelleborni fuelleborni	AB272235
28S	Strongyloides procyonis	AB205054
28S	Strongyloides ratti	U39490
28S	Strongyloides robustus	AB272232
28S	Strongyloides stercoralis	U38855
28S	Strongyloides stercoralis	U39489

28S	Strongyloides stercoralis	DQ145661
28S	Strongyloides stercoralis	AY294186
28S	Stegelleta sp. JB-75	DQ145656
28S	Subanguina chilensis	DQ328724
28S	Subanguina radicicola	DQ328721
28S	Syphacia obvelata	EF464554
28S	Tetrabothriostrongylus mackerrasae	AM039751
28S	Thonus circulifer	AY593038
28S	Thonus circulifer	AY593039
28S	Thonus minutus	AY593047
28S	Thonus minutus	AY593048
28S	Thonus sp. JH-2004	AY593040
28S	Thonus sp. JH-2004	AY593041
28S	Tricoma sp. 3P15K2	DQ077785
28S	Tylencholaimus cf. teres HHBM-2007a	EF207243
28S	Tylencholaimus mirabilis	AY593059
28S	Tylencholaimus mirabilis	AY593027
28S	Tylencholaimus mirabilis	EF207242
28S	Tylencholaimus sp. JH-2004	AY593060
28S	Tylencholaimus sp. JH-2004	AY593028
28S	Tylenchorhynchus claytoni	EU368588
28S	Tylenchorhynchus claytoni	EU368589
28S	Tylenchorhynchus dubius	EU368590
28S	Tylenchorhynchus dubius	DQ328707
28S	Tylenchorhynchus leviterminalis	EU368591
28S	Tylencholaimellus sp. JH-2004	AY593055
28S	Tylenchina sp. WY-460	EU018047
28S	Torynurus convolutus	AY292806
28S	Trophonema arenarium	AY780971
28S	Trichodorus cylindricus	AM180728
28S	Trichodorus primitivus	AM180729
28S	Trichodorus similis	DQ832183
28S	Trichodorus similis	AM180730
28S	Troglostrongylus wilsoni	AY292807
28S	Trichinella spiralis	AF342803
28S	Trophurus sculptus	DQ328709
28S	Trichostrongylus colubriformis	AM039743
28S	Teratorhabditis palmarum	EF990717
28S	Teratorhabditis synpapillata	AB269817
28S	Turbatrix aceti	AY294184
28S	uncultured Aphanolaimus sp.	DQ086654

28S	Uncinaria sp. 3677	AF217874
28S	Uncinaria sp. 3675	AF217882
28S	Uncinaria sp. 3679	AF217881
28S	Uncinaria sp. 3671	AF217880
28S	Uncinaria sp. 3672	AF217888
28S	Uncinaria sp. 3681	AF217883
28S	Uncinaria sp. 3688	AF217887
28S	Uncinaria sp. 3682	AF217884
28S	Uncinaria sp. 3676	AF217869
28S	Uncinaria sp. 3685	AF217870
28S	Uncinaria stenocephala	AF217867
28S	Viscosia sp. 3P6K2	DQ077751
28S	Viscosia sp. 1I14K2	DQ077779
28S	Xiphinema citricolum	DQ299491
28S	Xiphinema citricolum	DQ299492
28S	Xiphinema citricolum	DQ285668
28S	Xiphinema citricolum	DQ299494
28S	Xiphinema citricolum	DQ299490
28S	Xiphinema citricolum	DQ299493
28S	Xiphinema floridae	DQ299508
28S	Xiphinema floridae	DQ299509
28S	Xiphinema floridae	DQ299507
28S	Xiphinema floridae	DQ299510
28S	Xiphinema georgianum	DQ299502
28S	Xiphinema georgianum	DQ299498
28S	Xiphinema georgianum	DQ299495
28S	Xiphinema georgianum	DQ299500
28S	Xiphinema georgianum	DQ299497
28S	Xiphinema georgianum	DQ299501
28S	Xiphinema georgianum	DQ299499
28S	Xiphinema georgianum	DQ299496
28S	Xiphinema laevistriatum	DQ299506
28S	Xiphinema laevistriatum	DQ299503
28S	Xiphinema laevistriatum	DQ299505
28S	Xiphinema laevistriatum	DQ299504
28S	Xiphinema tarjanense	DQ299511
28S	Xyala sp. 3P11K2	DQ077761
28S	Zeldia punctata	AF147070
28S	Zeldia punctata	DQ145662
28S	Zeldia punctata	EU195988
28S	Zeldia sp. JB-118	DQ145633

28S	Zeldia sp. JB-140	DQ145663
28S	Zoniolaimus mawsonae	AM039730
cox1	Pellioditis marina	AM937225
cox1	Pellioditis marina	AM937224
cox1	Pellioditis marina	AM937222
cox1	Pellioditis marina	AM937220
cox1	Pellioditis marina	AM937217
cox1	Pellioditis marina	AM937210
