# Supplementary figures and images for: Moving towards a complete molecular framework of the Nematoda: a focus on the Enoplida and early-branching clades
Source: BMC Evol Biol. 2010 Nov 12;10:353. doi: 10.1186/1471-2148-10-353 (PMC2995457; doi:10.1186/1471-2148-10-353)

Figure S1

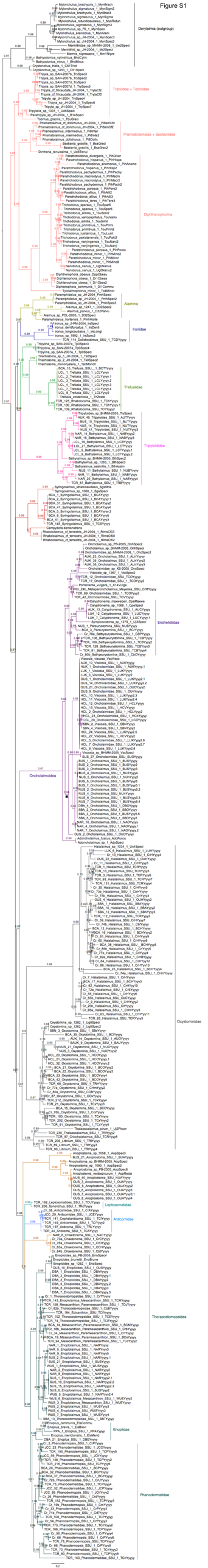

Figure S2

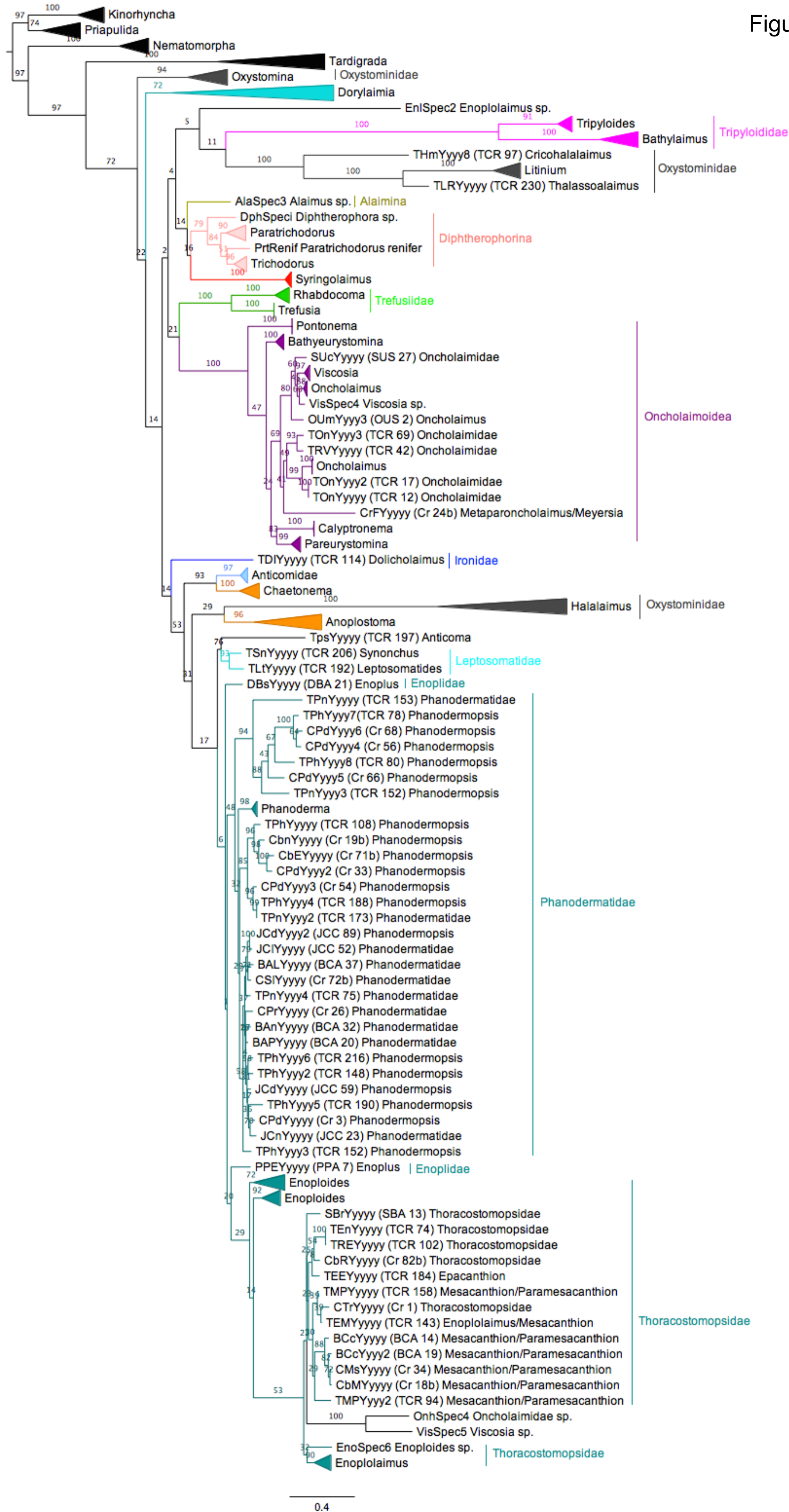

Figure S3

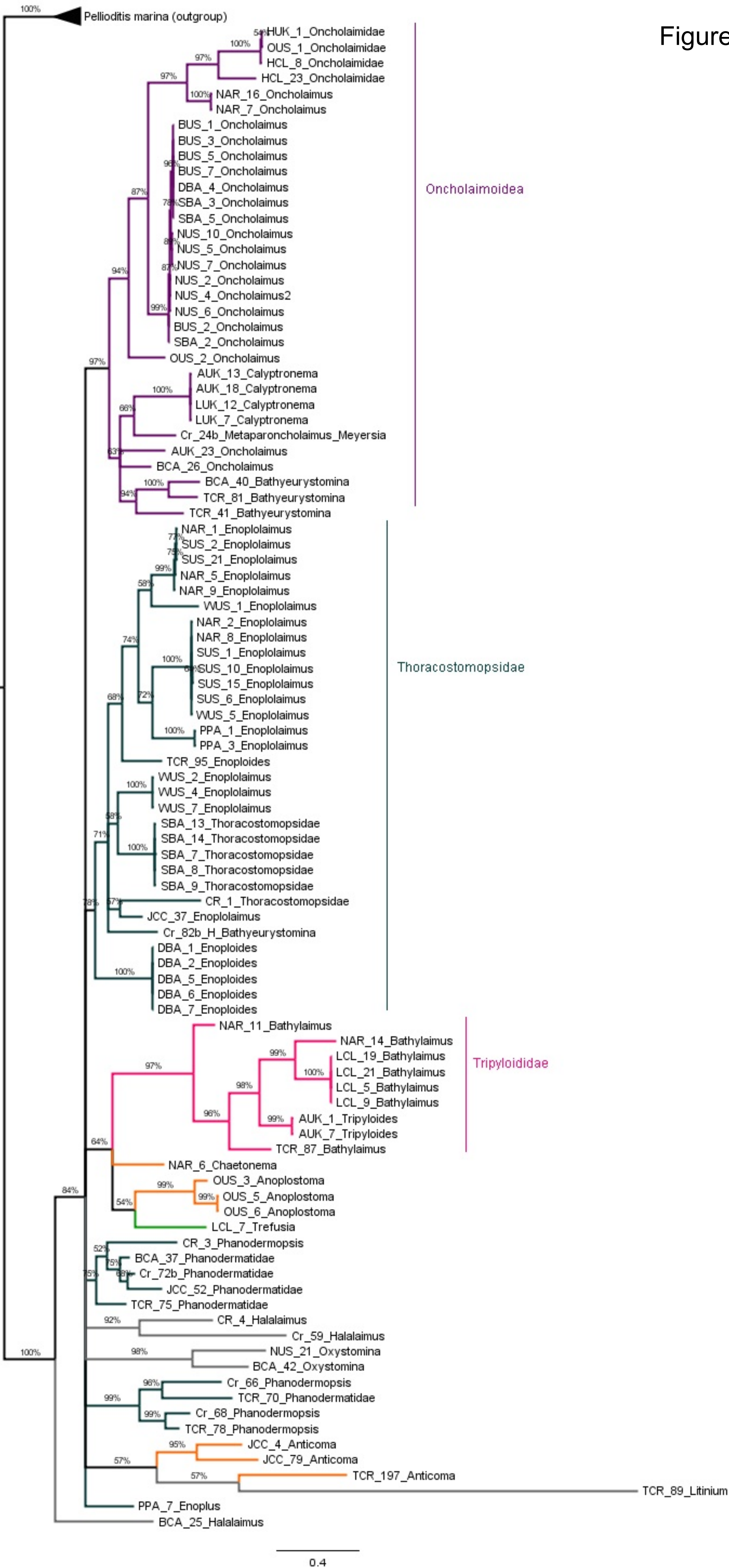

Supplement: Additional file 2 — Supplementary 18S, 28S and cox1 phylogenies. Figure S1: Expanded Maximum Likelihood 18S phylogeny of the Enoplia, fully expanded and annotated with taxonomic classifications. Tree built using 18S sequence data from 81 taxa, with estimation of the P-Invar parameter and partitions according to 18S secondary structure. The black square within Oncholaimoidea represent the primary shallow-water clades containing the majority of Oncholaimus and Viscosia specimens isolated in this study. Figure S2: Maximum Likelihood phylogeny of the nematode subclass Enoplia, built using sequences from the D2/D3 expansion region of the 28S gene. Tree constructed using 433 taxa, with estimation of P-Invar parameter. No alignment regions were excluded from the analysis. Figure S3: Bayesian phylogeny of the nematode order Enoplida built using cox1 gene sequences. Tree constructed using a 3 alignment partitions according to codon positions, using 2 million generations, and chain heating temperature of 0.1. [file 1471-2148-10-353-S2.PDF]
